# Integrated small RNA and mRNA expression profiles reveal miRNAs and their target genes in response to *Aspergillus flavus* growth in peanut seeds

**DOI:** 10.1186/s12870-020-02426-z

**Published:** 2020-05-13

**Authors:** Chuanzhi Zhao, Tingting Li, Yuhan Zhao, Baohong Zhang, Aiqin Li, Shuzhen Zhao, Lei Hou, Han Xia, Shoujin Fan, Jingjing Qiu, Pengcheng Li, Ye Zhang, Baozhu Guo, Xingjun Wang

**Affiliations:** 1 Biotechnology Research Center, Shandong Academy of Agricultural Sciences, Shandong Provincial Key Laboratory of Crop Genetic Improvement, Ecology and Physiology, Jinan, 250100 PR China; 2 College of Life Sciences, Shandong Normal University, Jinan, 250014 PR China; 3 Rizhao Experimental High School od Shandong, Rizhao, 276826 PR China; 4 Department of Biology, East Carolina University, Greenville, NC USA; 5 Crop Protection and Management Research Unit, USDA-Agricultural Research Service, Tifton, GA 31793 USA; 6 Department of Plant Pathology, University of Georgia, Tifton, GA USA

**Keywords:** Peanut, *Aspergillus flavus*, microRNA, Transcriptome, Degradome

## Abstract

**Background:**

MicroRNAs are important gene expression regulators in plants immune system. *Aspergillus flavus* is the most common causal agents of aflatoxin contamination in peanuts, but information on the function of miRNA in peanut-*A. flavus* interaction is lacking. In this study, the resistant cultivar (GT-C20) and susceptible cultivar (Tifrunner) were used to investigate regulatory roles of miRNAs in response to *A. flavus* growth*.*

**Results:**

A total of 30 miRNAs, 447 genes and 21 potential miRNA/mRNA pairs were differentially expressed significantly when treated with *A. flavus*. A total of 62 miRNAs, 451 genes and 44 potential miRNA/mRNA pairs exhibited differential expression profiles between two peanut varieties. Gene Ontology (GO) analysis showed that metabolic-process related GO terms were enriched. Kyoto Encyclopedia of Genes and Genomes (KEGG) pathway analyses further supported the GO results, in which many enriched pathways were related with biosynthesis and metabolism, such as biosynthesis of secondary metabolites and metabolic pathways. Correlation analysis of small RNA, transcriptome and degradome indicated that miR156/SPL pairs might regulate the accumulation of flavonoids in resistant and susceptible genotypes. The miR482/2118 family might regulate NBS-LRR gene which had the higher expression level in resistant genotype. These results provided useful information for further understanding the roles of miR156/157/SPL and miR482/2118/NBS-LRR pairs.

**Conclusions:**

Integration analysis of the transcriptome, miRNAome and degradome of resistant and susceptible peanut varieties were performed in this study. The knowledge gained will help to understand the roles of miRNAs of peanut in response to *A. flavus*.

## Background

Peanut (*Arachis hypogaea*. L), or groundnut, is one of the most important oil crops in the world. It is wildly planted in Asia, Africa, and North America. China, India and the United States are the world’s major producers of peanut. The world’s total volume of peanut production is about 29 million metric tons per year (http://www.peanutsusa.com), contributing to an estimated value of about $35 billion [1]. However, peanut production is vulnerable to the threat of aflatoxin contamination, which is caused by the infection of *Aspergillus flavus* and *Aspergillus parasiticus* [2]. The aflatoxin was first revealed in 1960, when the aflatoxin-contaminated peanut caused the death of a large number of turkeys in UK. Aflatoxins, can cause DNA damage and chromosome instability in host cells, are highly toxic and one of the most carcinogenic substances [3–5]. Peanut aflatoxin contamination is serious problem in China and limits the exportation of peanuts. Contamination can occur in the field, during harvest, or in storage and processing, and therefore, aflatoxin contamination is hard to prevent and control.

Although it is a widespread problem, the underlying molecular determinants and mechanisms of peanut-*A. flavus* interaction have remained elusive. The analysis of the molecular mechanism of peanut-fungus interactions, and mining peanut resistance information is the basis to develop resistance peanut varieties. To better understand the mechanisms, cDNA libraries derived from developing peanut seeds from a resistant cultivar (GT-C20) and a susceptible cultivar (Tifrunner) were constructed and a total of 21,777 EST sequences were generated [6]. Soon afterwards, a peanut oligonucleotide microarray chip was used to identify resistance genes to *A. flavus* and *A. parasiticus* mixed infection, and found that 62 genes in resistant cultivar were up-regulated and the expression of 22 putative *Aspergillus*-resistance genes were higher in the resistant cultivar in comparison to the susceptible cultivar [1]. In order to identify proteins related to the resistance to aflatoxin contamination, two-dimensional electrophoresis was employed with mass spectrometry as well as real time RT-PCR method and 12 potential different expressed proteins were identified [7]. Another group also used the differential proteomic approach and identified an array of proteins responding to *A. flavus* infection [8]. Recently, Wang et al. employed Next Generation Sequencing to survey the gene expression profiling of resistant and susceptible peanut genotypes in response to *A. flavus* growth [9, 10]. These studies provided valuable information for understanding the mechanism of peanut in response to aflatoxin production. However, due to the lack of peanut genomic sequences, it is hard for these studies to provide a comprehensive interpretation of the transcriptomic changes of peanut in response to *A. flavus*. Recently, the release of whole genome sequences of the diploid wild peanut species, *A. duranensis* and *A. ipaensis* [11, 12] and cultivars, Tifrunner and Shitouqi (https://peanutbase.org/, http://peanutgr.fafu.edu.cn/) provide new opportunity to understand the peanut resistance mechanism in response to *A. flavus*.

MicroRNAs (miRNAs) are a class of non-coding RNAs regulating gene expression and play roles in plant development and response to stresses [13–15]. Up to now, more than 5000 miRNAs have been identified from near 70 plant species (http://www.mirbase.org/). The functions of plant miRNAs in biotic stress responses have been extensively explored. For example, miR398 regulated plant productivity under oxidative stress conditions by targeting Cu/Zn superoxide dismutase [16, 17]. In tomato, the accumulation of miR169 was induced by drought stress. The expression of its target genes, nuclear factor Y subunit genes and multidrug resistance-associated protein gene, were significantly down-regulated by drought stress. Over-expression of miR169 enhanced drought tolerance of tomato [18, 19]. Many transcription factors, such as SPL [20, 21], MYB [22], NAC [23–25], ARF [26], HD-Zip [27] and AP2 [28, 29] were the targets of miRNAs, suggesting miRNAs located in the core position of the network in gene regulation.

Recently, the roles of miRNA in plant disease resistance were gradually discovered, and miRNAs were considered to play key roles in plant natural immune system [30, 31]. In *Arabidopsis*, it was proven that miR472/RDR6 silencing pathway modulated PAMP and effector triggered immunity (ETI) through the post-transcriptional control [32]. In rice, multiple miRNAs are involved in resistance against the blast fungus *Magnaporthe oryzae.* A group of rice miRNAs were differentially expressed upon *M. oryzae* infection. Over-expression of miR160a and miR398b enhanced resistance to *M. oryzae* in the transgenic rice [33]. Multiple miRNA target genes were related to disease resistance. For example, the expression and the variation of miR482 and miR2118 reflected the shift in the balance of plants NBS-LRR defense system [34]. In our previous studies, we identified many known and novel miRNAs in peanut using high-throughput sequencing method [35]. We found that the expression of some known and novel miRNAs was induced or inhibited upon *Ralstonia solanacearum* infection in cultivated and wild peanuts. Degradome sequencing results possibly indicated that some defense response genes were degraded by miRNA [36]. These data suggested that miRNA might play important role in peanut disease resistance.

In this study, we aimed to identify potential *A. flavus* responsive miRNAs and their targets in peanut, and study their roles in peanut-*A. flavus* interaction. For this purpose, we employed high-throughput sequencing technology to simultaneously analyze the miRNA and mRNA expression profiles and their regulation in response to *A. flavus* growth using a resistant and a susceptible peanut variety. Our results provided valuable information for understanding the molecular mechanism of peanut resistance to *A. flavus*.

## Results

### Different resistance of peanut varieties GT-C20 and Tifrunner to *A. flavus*

**Fig. 1:**
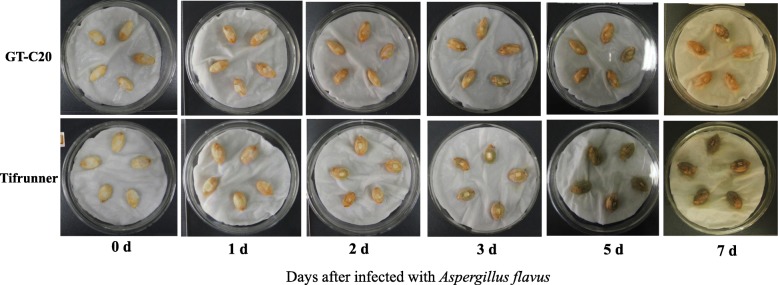
Phenotype of peanut variety GT-C20 and Tifrunner after infected with *Aspergillus flavus*

The resistance of peanut varieties GT-C20 and Tifrunner to *A. flavus* was validated. As a result, in two days after inoculation (DAI), hyphae were only observed in the seeds of Tifrunner, but not in GT-C20 (Fig. 1). From three DAI, the hyphae were very clear in both GT-C20 and Tifrunner. More hyphae were observed in the seeds of Tifrunner than that in GT-C20 (Fig. 1). Statistical analysis showed that GT-C20 was high resistant (Infection index of 4.7) and Tifrunner was high susceptible (Infection index of 95.3) to *A. flavus* growth according to the standard reported [37]. Samples of two DAI were collected for miRNAome and transcriptome analysis.

### High-throughput sequencing and identification of miRNAs

**Fig. 2:**
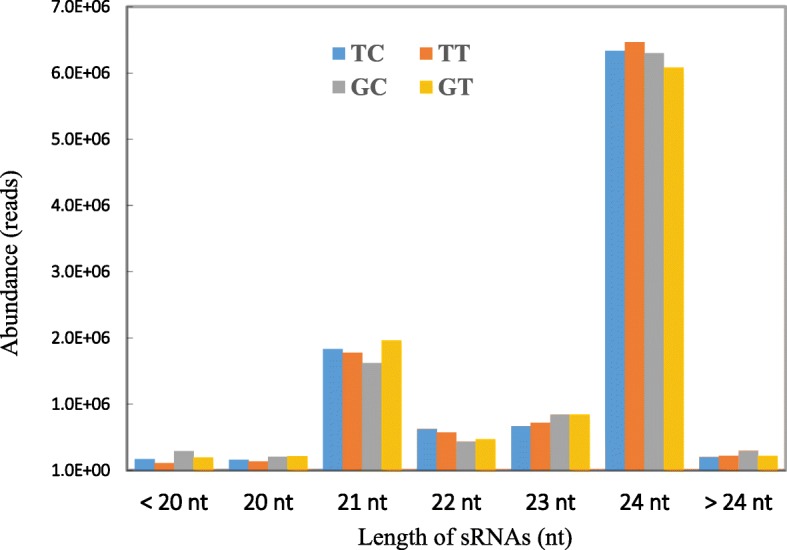
Sequence length distribution of small RNA in different libraries of peanut

To study the role of peanut miRNAs in response to *A. flavus* growth, eight small RNA libraries were constructed and sequenced using Illumina HiSeq2000 platform. These small RNA libraries included two *A. flavus*-treated (GT1 and GT2) and two control (GC1 and GC2) libraries of resistant cultivar GT-C20, and two *A. flavus*-inoculated (TT1 and TT2) and two control (TC1 and TC2) libraries of susceptible cultivar Tifrunner. After sequencing, a total of 111,839,986 raw reads and 111,324,210 clean reads were obtained from eight libraries (Additional file: Table S1). The normalized clean reads were used for analysis of small RNA distribution. Most of the clean reads were 20–24 nucleotides (nt) in length, which accounted for 96.22, 96.67, 94.06 and 95.81% of all clean reads in TC, TT, GC and GT libraries, respectively (Fig. 2). The 24-nt small RNAs was the most abundant group, which was followed by the 21-nt small RNAs. These results were consistent with previous studies in peanut small RNA sequencing [35, 36, 38].

More than 85.5% unique small RNAs and 86% total small RNAs were mapped to peanut genome (*A. duranensis* and *A. ipaensis*, https://peanutbase.org/) using SOAP2 software (Additional file: Table S2). By blast search against the miRNA database (miRbase 21.0, http://www.mirbase.org/), we identified 113 known miRNAs, belongs to 30 miRNA families (Additional file: Table S3). In addition, clean small RNAs were also used to blast against with GenBank and Rfam database to remove rRNAs, tRNAs, snoRNAs and snRNAs (Additional file: Figure S1). The other small RNAs were used to explore novel miRNAs according the criteria described by the previous studies [35, 39, 40]. In total, we identified 67 novel miRNAs, ranging from 20 nt to 23 nt, and the negative folding free energies of their precursor’s hairpin structures ranged from 184.8 to 19.1 kcal/mol (Additional file: Table S4).

### Expression of miRNAs in different peanut varieties response to *A. flavus* infection

**Fig. 3:**
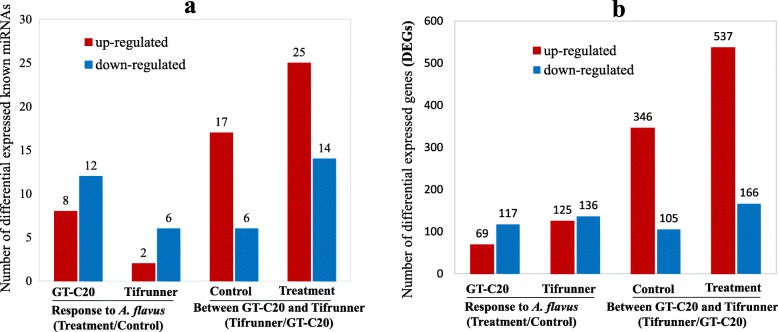
Numbers of differentially expressed known miRNAs (**a**) and genes (**b**) in response to *A. flavus*

**Fig. 4:**
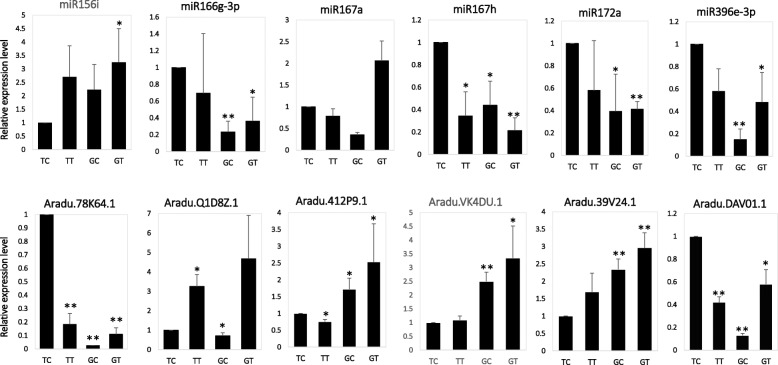
Validation of the relative expression level of partial miRNA and mRNA by qRT-PCR. Error bars indicate ± SE obtained from three biological repeats. Student’s T-test was performed to analyze the changes in the gene expression after treated with *A. flavus*. **denotes the *p* value < 0.01 and *denotes the *p* value < 0.05

To identify miRNAs in peanut that respond to *A. flavus*, the differential expressed miRNAs were identified at a significant level (adjusted *p*-value < 0.05 and fold change ≥1.5) by comparing the normalized miRNA expression level using TP10M (Tags per ten million). Among the 113 known miRNAs identified in peanut, eight and two were up-regulated, twelve and six were down-regulated in response to *A. flavus* in GT-C20 and Tifrunner, respectively (Fig. 3a, Additional file: Table S3). The total number of differentially expressed known miRNAs in GT-C20 (20) was more than that in Tifrunner (8). Among these differentially expressed known miRNAs, the expression of miR319 were both increased in GT-C20 and Tifrunner after *A. flavus* inoculation. While the expression of miR157a and miR157d were both decreased in GT-C20 and Tifrunner after treated with *A. flavus*. In addition, miR157a showed the highest expression abundance in all known miRNAs (Additional file: Table S3). There were also some miRNAs showing different expression trend in response to *A. flavus* in different peanut varieties. For example, the expression of miR156a was increased in GT-C20 in response to *A. flavus*, but unchanged in Tifrunner (Additional file: Table S3). Moreover, twelve differentially expressed novel miRNAs were identified in response to *A. flavus*, including six novel miRNAs from GT-C20 and six novel miRNAs from Tifrunner (Additional file: Table S4). In GT-C20, miRn21, miRn56 and miRn67 were induced or specifically expressed in response to *A. flavus*, and miRn3, miRn9, miRn18 were depressed in response to *A. flavus*. In Tifrunner, miRn12, miRn21 and miRn25 were induced or specifically expressed in response to *A. flavus*, and miRn1, miRn53 and miRn55 were depressed in response to *A. flavus* (Additional file: Table S4). To confirm the sequencing results, six miRNAs (miR156i, miR166g-3p, miR167a, miR167h, miR172a and miR396e-3p) were selected for stem-loop qRT-PCR analysis (Fig. 4). The expression profiles of these miRNAs were in accordance with the deep sequencing data (Additional file: Figure S2).

The miRNA abundance between two different varieties was compared. Before inoculation, the abundance of all 113 known miRNAs of GT-C20 was 245,769 (TP10M), which is lower than that in Tifrunner (615,962). After inoculation, the abundance of all 113 known miRNAs in GT-C20 (269,075.54 TP10M) was also lower than that in Tifrunner (442,962.68 TP10M) (Additional file: Table S3). Under the criterion of adjusted *p* value (padj) < 0.05 and fold change ≥1.5, a total of 23 and 39 differential expressed known miRNAs between two varieties were identified in control and *A. flavus* infected samples, respectively (Fig. 3a). Many known miRNAs showed higher expression level in Tifrunner than in GT-C20 in both control and *A. flavus* infected samples. For examples, in control samples, the expression level of miR157a was 52,711 TP10M in GT-C20 and 291,784 TP10M in Tifrunner. And in treatment samples, the expression level of miR157a-5p was 32,146 TP10M in GT-C20 and 154,326 TP10M in Tifrunner (Additional file: Table S3). In addition, the expression level of miR2118 and miR482a in GT-C20 was also lower than that in Tifrunner (Additional file: Table S3).

### Targets identification of miRNAs by high throughout degradome analysis

To validate the targets of peanut miRNAs, a degradome pool was mixed with equal samples from TT, TC, GC and GT. Through high throughput sequencing, 14,338,349 raw reads representing 5,477,891 unique reads were obtained. All reads were used to align against the peanut genome and transcriptome using SOAP2 Program. As a result, 9,580,557 (66.82%) and 6,271,065 (43.73%) reads were successfully mapped in peanut reference genome and transcriptome, respectively (Additional file: Table S5). According to the signature number and abundance of putative cleaved position at each occupied transcript, these cleaved transcripts could be categorized into five classes according to the signature abundance at each occupied transcript position (0, 1, 2, 3 and 4) [41–43]. In our dataset, 249, 165, 554, 175 and 402 candidate targets were classified into categories 0, 1, 2, 3 and 4, respectively (Additional file: Table S6). In total, 93 and 176 candidate targets were identified for known and novel miRNAs, respectively (Additional file: Table S7-S8). We found many defense related genes were likely cleaved by miRNAs. For example, two PPR repeat-containing protein genes, Aradu.7H0DM.1 and Aradu.4L72B.1 were potentially cleaved by miR3514 and miRn10, respectively (Additional file: Table S8).

### Global mRNA expression profiles in peanut in response to *A. flavus* growth

To study the target gene expression changes in response to *A. flavus* growth, eight transcriptome libraries were constructed using the same samples as used in miRNA study. Through transcriptome sequencing, a total of 97,021,101 raw reads were generated with an average of ∼12 million reads per libraries (Additional file: Table S9). Approximately 77.83% reads were successful mapped to the peanut reference genome, and about 8.18% reads were mapped to multiple regions (Additional file: Table S9). In peanut genome database, 40,636 genes from *A. duranensis* (A genome) and 46,984 genes from *A. ipaensis* (B genome) were deposited. In our RNA-seq data, 30,254 (74.45%) genes of *A. duranensis* and 34,172 (72.73%) genes of *A. ipaensis* were mapped, indicating that these genes were expressed in peanut seeds.

### Differentially expressed genes in response to *A. flavus*

**Fig. 5:**
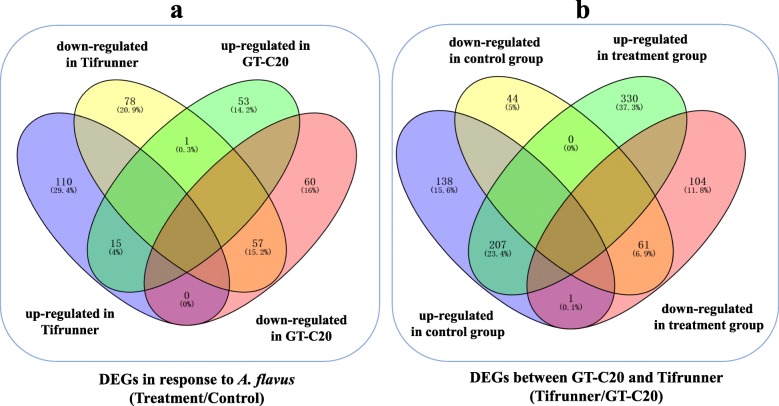
Venn diagram of differentially expressed genes in response to *A. flavus* (**a**) and between two peanut varieities (**b**). Venny analysis was performed using the online software VENNY^2.1^ (http://bioinfogp.cnb.csic.es/tools/venny/index.html)

We further investigated the global gene expression profile of peanut in response to *A. flavus*. By applying a cut-off criterion of probability ≥0.8 and |log_2_(fold change) | ≥ 1, a total of 261 (125 up-regulated and 136 down-regulated) and 186 (69 up-regulated and 117 down-regulated) differentially expressed genes (DEGs) were identified in response to *A. flavus* from Tifrunner and GT-C20, respectively (Fig. 3b, Additional file Figure S3). Among these DEGs in response to *A. flavus*, 15 DEGs were up-regulated in both GT-C20 and Tifrunner, and 57 DEGs were down-regulated in both GT-C20 and Tifrunner (Fig. 5a). In addition, functional annotation results showed that 139 out of 447 (31.09%) DEGs were defense related genes, such as Chitinase, Heat shock protein, WRKY family transcription factor and temperature-induced lipocalin et al. (Table 1). Moreover, we found that an Indole-3-acetic acid inducible 2 gene (Araip.AN5V8.1) showed opposite expression trend in response to *A. flavus*. It was up-regulated in resistant variety GT-C20, but down-regulated in susceptible variety Tifrunner (Fig. 5a). The qRT-PCR analysis for several DEGs (Aradu.78 K64.1, Aradu.Q1D8Z.1, Aradu.412P9.1, Aradu.VK4DU.1, Aradu.39 V24.1, and Aradu.DAV01.1) confirmed the RNA-seq results (Fig. 4, Additional file Figure S2).

**Table 1:** Differential expressed defense related genes in response to *A. flavus*

Genes annotation	No. of Genes	Expression trend		Gene list
		Tifrunner	GT-C20	
Aluminium induced protein with YGL and LRDR motifs	3	down	down	Aradu.04RDY, Araip.FVH8I, Araip.ZX8HU
Autotransporter adhesin	1	down	down	Araip.52YBX
Auxin-responsive protein	2	up	up	Aradu.GIN82, Araip.XVL9X
Expansin 1	2	up	up	Aradu.DSS3T, Araip.IN0BK
Cellulose synthase like E1	2	up	–	Aradu.BZH82, Araip.FL29A
Chalcone synthase	10	up	up	Aradu.72KEV, Aradu.9BM9J, Araip.Z5UEI, Aradu.J6XSM, Aradu.JL5FQ, Aradu.LZ0RH, Aradu.XCU6I, Aradu.ZWT01, Araip.3T4SK,Araip.B8TJ0, Araip.E7BUX
Chitinase A	1	–	up	Araip.8C3IU
Copper amine oxidase	2	–	up	Aradu.NCJ0H, Araip.26B5V
Cytochrome P450	18	up	up	Aradu.0I3GY, Aradu.4262 U, Aradu.5F6FU, Aradu.73MTJ, Aradu.9F1DZ, Aradu.A7CMV, Aradu.MI3AU, Aradu.Q1D8Z, Aradu.RZ1DG, Aradu.TJ0ZU, Araip.0P3RJ, Araip.B1BRC, Araip.B5SML, Araip.D77W5, Araip.N872L, Araip.RXK1S, Araip.S5EJ7, Araip.T8GZM
Disease resistance protein	14	up	up	Aradu68L7, Araip.VGW7F, Aradu.CHQ37, Aradu.KI3UZ, Aradu.ZH6BL, Araip.23EKN, Araip.D2NS0, Araip.H1IIW, Araip.J0C95, Araip.JPJ83, Araip.MQ6CB, Araip.TSU7Y, Araip.W3N2F, Araip.Z0AKG
DNAJ-like	2	down	down	Araip.59BNM, Aradu.D1YQE
Early nodulin-related gene	2	down	down	Aradu.X9GQ3, AraipL9GW
Ethphon-induced protein	1	down	down	Aradu.X5SR9
Ethylene-responsive transcription factor	2	up	up	Aradu.B90GQ, Araip.T3D3V
Ethylene-responsive transcription factor	4	down	down	Aradu.GB4U4, Araip.3JJ8N, Aradu.NZ8CP., Araip.LJJ47
ExpansinB	3	down	down	Aradu.WXM55, Aradu.MR104, Araip.0US7S
Ferritin 4	4	down	down	Aradu.N8FJN, Aradu.W4RCV, Araip.46XVA, Araip.RJ07Z
Glutathione S-transferase	3	down	down	Aradu.6PF06, Aradu.H8WP2, Araip.J9Q6I
Heat shock protein	4	down	down	Aradu.A3TK2, Araip.G7QFC, Aradu.NE0BE, Aradu.JL6EF
Indole-3-acetic acid inducible 2-gene	1	down	up	Araip.AN5V8.1
Late embryogenesis abundant (LEA) protein	6	up	up	Aradu.60I66, Aradu.8S28F, Aradu.TW8M6, AraipJG8Y, Araip.4NS5K, Araip.DF76F
Late embryogenesis abundant (LEA) protein	2	down	down	Aradu.CLY7T, Araip.VM8FV
Lipase/lipooxygenase (LOX)	2	down	–	Aradu.MAS03, Araip.081EX
MYB transcription factor	2	up	up	Aradu.CT448, Araip.VH6HT
Nitrate transporter	2	up	up	Aradu.BDD78, Aradu.GZK47
Nodulin MtN21	3	down	down	Aradu.TY9X1, Araip.AV3G9, Araip.HXZ5T
O-methyltransferase 1	9	up	up	Aradu.FEK42, Araip.E3E4E, Aradu.RW4KA, Aradu.97Y2Q, Araip.Z3XZX, Araip.6K01Z, Aradu.0H1MY, Aradu.C09GA, Aradu.Y6TV9
Pectinesterase	2	down	–	Aradu.S6DQM, Araip.EQZ9W
Peroxidase	2	down	down	Aradu.2BI47, Araip.BKI6W
Peroxidase	2	–	up	Aradu.BNR06, Araip.595JK
Phosphate-responsive gene	1	down	down	AraduX84T
Calcium-binding protein CML25-like	2	up	up	Aradu.82C4A, Araip.HQ67P
Receptor-like kinase	1	up	up	Araip.KAF3M
Receptor-like kinase	4	down	down	Aradu.HZ14S, Araip.9ND7T, Aradu.AH39E, Araip.HQ67P
Senescence-associated family protein	10	down	down	Araip.EBV68, Aradu.RPS70, Araip.82RV8, Araip.T1QD3, Aradu.T1NFP, Araip.0B03Z, Araip.97YGM, Araip.GPA1K, Araip.HM6LF, Araip.W2CG9
Stress induced protein;	2	down	down	Araip.7G428, Araip.K8M87
Temperature-induced lipocalin	2	down	down	Aradu.Y28R7, Araip.YA4GL
Thioredoxin superfamily protein	2	down	down	Araip.H56DJ, Aradu.TES1U
WRKY family transcription factor	2	up	–	Aradu.S7YD6, Araip.RC4R7

### Differentially expressed genes between resistant and susceptible peanut varieties

We further analyzed the gene expression variation between two peanut varieties. By comparison with control samples (GC and TC), 451 DEGs (346 up-regulated and 105 down-regulated) were identified (Fig. 3b). For treatment samples (GT and TT), 703 DEGs (537 up-regulated and 166 down-regulated) were identified (Fig. 3b). Some disease related genes showed different expression level in two varieties. For example, one NBS-LRR disease resistance gene (Araip.WF303.1, log_2_ TC/GC = 9.47, log_2_ TT/GT = 4.66) was highly expressed in Tifrunner, but had a lower expression level in GT-C20. Meanwhile, another NBS-LRR encoding gene (Aradu.168 L7.1) showed the opposite expression trend, highly expressed in GT-C20, but had a lower expression level in Tifrunner (Fig. 3b).

### GO and KEGG pathways of DEGs

**Fig. 6:**
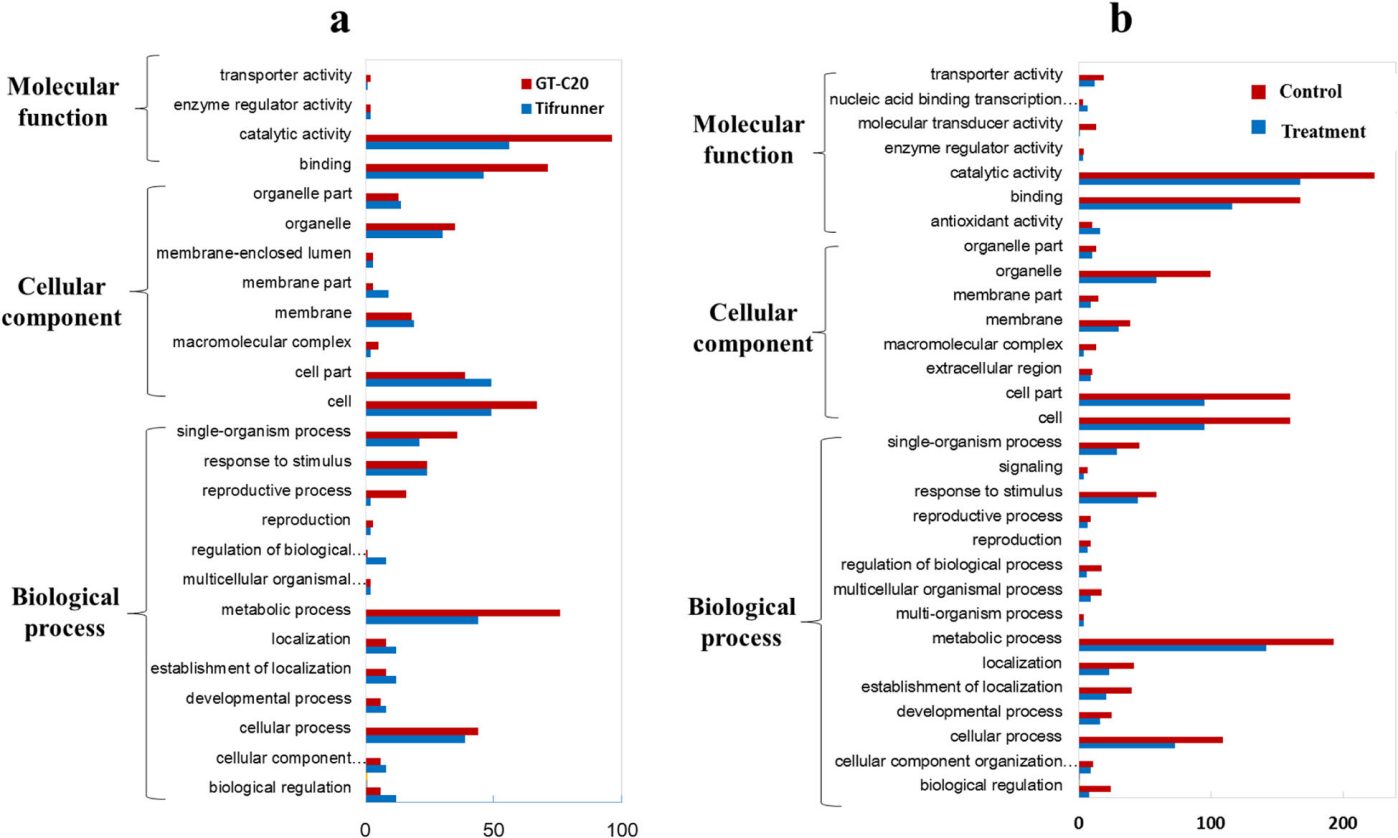
GO analysis of differentially expressed genes. **a** DEGs in response to *A. flavus*, **b** DEGs between Tifrunner and GT-C20. Gene are classified into three main categories: biological process, cellular component, and molecular function. The x-axis indicates the number of genes in a category, and the y-axis means the GO tems of genes

GO analysis of the DEGs was performed and DEGs could be classified into three categories including molecular function, cellular component and biological process. In category of cellular component, “cell” and “cell part” represented the top terms. For the category of molecular function, the GO term of “catalytic activity” was enriched. For the category of biological processes, the most abundant terms were “metabolic process” and “cellular process” (Fig. 6a). The DEGs between control samples and treatments were also analyzed. Two peanut varieties have difference in many GO terms in both control samples and treatment samples, such as “metabolic process”, “catalytic activity” and “cellular process” (Fig. 6b).

**Fig. 7:**
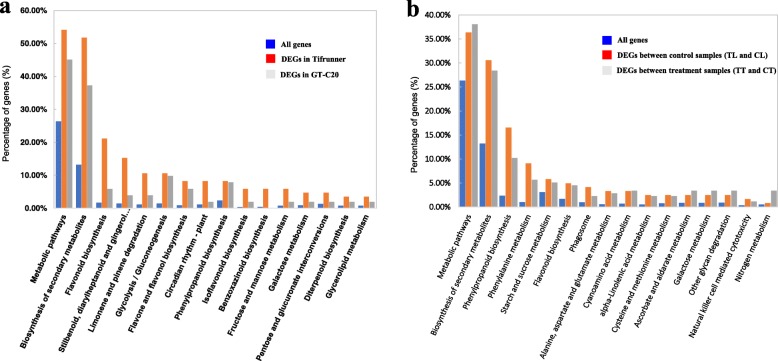
KEGG enrichment analysis of differentially expressed genes. **a** KEGG enriched pathways in response to *A. flavus*; **b** KEGG enriched pathways between Tifrunner and GT-C20

To better understand the function and gene regulatory network, KEGG analysis was carried out. The DEGs in response to *A. flavus* were assigned into 53 and 41 KEGG pathways in Tifrunner and GT-C20, respectively (Fig. 7, Table S10–11). The top 16 enriched pathways possibly regulated by *A. flavus* were summarized (Fig. 7a, Table S10). Most enriched pathways were related to biosynthesis and metabolism, such as “biosynthesis of secondary metabolites”, “flavonoid biosynthesis”, “stilbenoid, diarylheptanoid”, “gingerol biosynthesis”, “metabolic pathways”, and “limonene and pinene degradation” etc. Interestingly, we observed that, in most of the enriched pathways including “biosynthesis of secondary metabolites” and “metabolic pathways”, the numbers of DEGs in Tifrunner was more than that in GT-C20, indicating the difference in metabolic active between two varieties. Interestingly, several pathways of biosynthesis and metabolism were also enriched, such as “biosynthesis of secondary metabolites”, “phenylpropanoid biosynthesis”, “phenylalanine metabolism”, “metabolic pathways”, “flavonoid biosynthesis” and “ascorbate and aldarate metabolism” etc. KEGG results also supported the difference in the metabolic activity between two peanut varieties (Fig. 7b, Table S11).

### Correlation analysis of miRNAs expression profiles and their target genes

To specify the roles of miRNAs in response to *A. flavus* growth, the expression profiles of miRNAs and targets were analyzed. In response to *A. flavus* infection, twenty-two potential miRNAs/targets pairs were identified (Table S12). Degradome sequencing showed these target genes were cleaved by miRNAs. However, the expression profiles of miRNAs were not perfectly negative corresponding to these targets. For example, only five miRNAs/targets pairs showed one-to-one correspondence in Tifrunner, including five up-regulated miRNAs/down-regulated targets and two down-regulated miRNAs/up-regulated targets in response to *A. flavus* growth. In addition, five miRNAs/targets pairs showed opposite expression trend in Tifrunner after *A. flavus* infection. For other miRNAs/targets pairs in Tifrunner, only the miRNAs or targets showed differential expression in response to *A. flavus*, and the one-to-one correspondence expression was not observed.

Between two peanut varieties, forty-four differentially expressed miRNAs/targets pairs were identified (Table S13). We further compared the one-to-one expression correspondence of miRNAs/targets pairs between two peanut varieties. Between control samples and *A. flavus* infected samples (GC and TC), differentially expressed miRNAs and targets were simultaneously observed only in 16 miRNAs/targets pairs, and these results were not observed in other miRNAs/targets pairs. Among these 16 miRNAs/targets pairs, the expression of miRNA and targets were negatively correlated. For the rest five miRNAs/targets, the expression trend of miRNA and target was in the same trend, both up-regulated or down-regulated. For the *flavus* infected samples (GT and TT), differentially expressed miRNAs and targets were simultaneously observed in 14 miRNAs/targets pairs, ten of them showed negative correlation in expression of miRNAs and targets.

## Discussion

### Strategies to reveal the disease resistant mechanism of peanut

Cultivated peanut (*Arachis hypogaea*. L) is an allotetraploid (2n = 4x = 40, AABB) organism, which emerged as a hybridization of two ancient diploid species, probably *A. duranensis* (A-genome) and *A. ipaensis* (B-genome), followed by spontaneous whole genome duplication [44–46]. Expressed Sequence Tag (EST) was first used to obtain gene sequence information and their response to biotic and abiotic stresses in peanut [47–49]. Based on EST sequencing and Microarray technology, dozens of different genes response to *A. flavus* infection were identified, which provide a useful information for understanding the molecular mechanism of peanut resistance to *A. flavus* [1, 6]*.* Recently, the whole genome sequencing of two diploid wild peanut species has been completed (http://peanutbase.org/). This achievement will greatly promote gene discovery and genetic improvement of this major crop. Here, we employed the high throughput mRNA and miRNA sequencing to investigate the gene expression and regulation of peanut upon *A. flavus* infection. Our data covered the expression information of more than 64,426 genes. It is the first report of genome-wide gene expression profiling study of peanut in response to *A. flavus*. Moreover, the integrated analysis of mRNAs and miRNAs might provide insight to understanding the miRNA regulatory in peanut in regulation of the resistance to *A. flavus.*

### Differential expression of defense related genes in resistant and susceptible peanut lines

In the present study, a total of 447 differentially expressed genes (DEGs) were identified in GT-C20 and Tifrunner in response to *A. flavus* (Fig. 4). Functional annotation results showed that 139 of these genes were defense related genes (Table 1). The majority of defense related DEGs (132 out of 139) were differentially expressed in both peanut varieties, while only seven of the DEGs were differentially expressed only in one peanut variety in response to *A. flavus*.

Chitin is a key component of fungal cell wall and is considered as an essential factor for maintaining the pathogenicity of the fungus. Chitinases are hydrolytic enzymes that have the activity of degrading chitin. Accumulating evidence indicated that the activity of chitinase was important for plant resistance against pathogens infection [50–52], and some chitinases were pathogenesis related (PR) proteins [53–55]. We observed that the expression of a chitinase gene (Araip.8C3IU.1) had no change in Tifrunner but was induced in GT-C20. In addition, our data showed that two peroxidases (Aradu.BNR06.1 and Araip.595JK.1) and two copper amine oxidases (Aradu.NCJ0H.1 and Araip.26B5V.1 were up-regulated in GT-C20 but no significant expression difference in Tifrunner (Table 1). Interestingly, peroxidase and blue copper protein genes were also disease related genes [56, 57]. In plant, peroxidases were involved in plant disease resistance response through participating biosynthesis of bactericide and lignin. Copper amine oxidases contribute to catalyze the oxidative de-amination of polyamines and producing hydrogen peroxide (H_2_O_2_). In plants, H_2_O_2_ derived from polyamine oxidation mediated cell death and was correlated with stress response to pathogen invasion [58, 59]. Plant cell walls were considered as the first security system barrier of defense against bacterial and fungal pathogen. Pectin is an important component in plant cell wall. In plants, pectinesterase/pectinesterase inhibitor acts to modify the cell walls via degration of pectin. In our dataset, two pectinesterase/pectinesterase inhibitor genes (Aradu.S6DQM.1 and Araip.EQZ9W.1) were significantly depressed in Tifrunner but no significant expression difference in response to *A. flavus* (Table 1). Here, the difference expression trend of these genes in resistant and susceptible peanut varieties provided us a useful clue to reveal the molecular mechanism of peanut response to *A. flavus*.

Among these 139 defense related DEGs, 18 genes encode cytochrome P450 superfamily genes (Table 1). As a multi-functional gene, the function of cytochrome P450 in defense reaction were revealed in many plants [60, 61]. In rice, over-expressing of *CYP71Z2* enhanced resistance to bacterial blight. Further studies confirmed that cytochrome P450 gene contributes to enhance rice disease resistance to pathogens both through mediating diterpenoid phytoalexin accumulation and via suppression of IAA signaling in rice [62].

NBS-LRR genes represent one of the largest disease resistance gene families in plants [63]. So far, the majority of the disease-resistant genes identified by map-based cloning were NBS-LRR genes, such as resistance to rice blast [64], powdery mildew resistant genes *Pm3/Pm8* in wheat [65], leaf rust resistance gene *Lr10* in wheat [66]. We found two NBS-NBS-LRR type disease resistance genes (Aradu.168 L7.1, Araip.VGW7F.1) were induced in both peanut varieties. However, the increased expression level of the two genes were more dramatic in resistant genotype than that of susceptible genotype peanut. For example, the expression level of Aradu.168 L7.1 was almost equal in both varieties in control samples (log_2_^TC/GC^ = 0.11). In response to *A. flavus*, the expression of this gene was induced in both resistant (log_2_^GT/GC^ = 3.28) and susceptible peanut (log_2_^TT/TC^ = 0.68) genotype peanuts. After treatment with *A. flavus*, the expression of this Aradu.168 L7.1 in resistant genotype was significantly higher than that in susceptible genotype (log_2_^GT/TT^ = 2.72). Araip.VGW7F.1 had the same expression patterns with that of Aradu.168 L7.1, which was induced in both peanut varieties but were more dramatically induced in resistant genotype (log_2_^GT/TT^ = 2.84). Aradu.168 L7.1 and Araip.VGW7F.1 located in chromosome A05 (1880820–1,893,586) and B05 (1716985–1,720,472) of peanut, respectively, and were considered as orthologous genes. Interestingly, sequence alignment results showed that Aradu.168 L7.1 (Expect: 5e-146, Identities: 316/943(34%) and Positives: 506/943(53%)) and Araip.VGW7F.1 (Expect: 2e-119, Identities: 292/931(31%) and Positives: 490/931(52%)) were all homologous with the disease resistance locus, *RPM1*, of Arabidopsis. The *RPM1* locus conferred resistance to the bacterial pathogen *Pseudomonas syringae* in Arabidopsis [67, 68] and was supposed to have functional conservation in bean, pea and other crop species [69].

Besides, other several defense related genes also showed differential expression profile between two different peanut genotypes including two cellulose synthase like E1 genes, two WRKY family transcription factor genes, two lipase/lipooxygenase (LOX) genes and one indole-3-acetic acid inducible 2 gene (Table 1). WRKY represents one of largest transcription factor gene families. Some WRKY gene members have been reported in regulating response to various biotic and abiotic stresses. Here, we found that two WRKY genes, Aradu.S7YD6 and Araip.RC4R7, located on chromosome A06 and B06, respectively, were homologous with *WRKY33* genes of other plants. In *Arabidopsis, WRKY33* was required for resistance to necrotrophic fungal pathogens. In our dataset, the above two WRKY genes were up-regulated in Tifrunner but remained unchanged in GT-C20 which seemed to be the opposite of what was expected in other plants. In addition, we found that two cellulose synthase like E1 genes (Aradu.BZH82, Araip.FL29A) were up-regulated in Tifrunner but did not showed significant differential expression in GT-C20 in response to *A. flavus.* Cell wall is an important barrier for plants against fungi which also seemed to be the opposite of what was expected in other plants*.* Previous studies have showed that lipoxygenase pathway was associated with the seed resistance against *A. flavus* in soybean and maize. For example, inactivation of the *ZmLOX3* could increases susceptibility of maize to *Aspergillus*. Here, two other lipase/lipooxygenase (LOX) genes, Aradu.MAS03 and Araip.081EX were down regulated in the Tifrunner but unchanged in GT-C20. In addition, indole-3-acetic acid inducible genes were involved in response to auxin stimulus and lateral root morphogenesis in other plants. Here, we found that an indole-3-acetic acid inducible 2 gene (Araip.AN5V8.1) was upregulated in the resistant variety and downregulated in the susceptible*.*

### Regulation of miRNAs in peanut response to *A. flavus*

Due to the sequence similarity, miR2118 was considered as member of miR482 superfamily [34, 70]. Emerging evidence indicated that miR482 and miR2118 both target the P-loop sequence motif of NBS-LRR defense genes in plants, and played a negative regulatory role in plant response to diseases [71]. In soybean, constitutive expression of miR482 led to enhance degradation of targeted R genes, resulting in increased nodulation [72]. In potato, the expression of miR482e was down-regulated in response to *Verticillium dahliae* infection which resulted in the up-regulation of its NBS-LRR targets. Overexpression of miR482e induced the decrease expression of its NBS-LRR targets, and the transgenic plantlets were highly susceptible to *V. dahlia* infection [73]. In our datasets, one member of miR2118 and three members of miR482 were identified. We found the overall expression level of miR2118, miR482a, miR482b and miR482b were lower in resistant genotype than in susceptible genotype (Table S3). For example, in resistant genotype, the expression abundance of miR2118 was 74.10 and 91.53 TP10M in mock and treatment samples, respectively. While in susceptible genotype, the expression abundance of miR2118 was 170.22 and 261.62 TP10M in mock and treatment samples, respectively (Table S3). Interestingly, peanut miR482/2118 miRNA family also target NBS-LRR defense genes including Aradu.168 L7, which has the higher expression level in resistant genotype than that in susceptible genotype.

In this study, 66 potential miRNA/targets pairs were identified through a combined analysis of the datasets of small RNA, degradome sequences and mRNA expression profiling. Among them, many SPLs (SQUAMOSA promoter-binding protein-like) might be regulated by miR156/157 miRNA family (Table S12–13). For example, Aradu.0GH1S.1 encodes a SPL transcription factor, which is homologous with *SPL12* of soybean (LOC100781289) and *SPL2* of Arabidopsis (AT5G43270). Degradome sequencing showed that Aradu.0GH1S.1 was potentially cleaved by many miRNA members of miR156/157 family, such as miR156a, miR156e and miR156i, etc. (Table S7). Previous evidences showed that *SPL* negatively regulated the accumulation of anthocyanin. And further studies proved that miR156/*SPL* regulated the secondary metabolism and the content of anthocyanin and flavonol in plants [74]. Both anthocyanin and flavonol were important flavonoids. Flavonoids have been shown to have antimicrobial properties against invading microorganisms including *A. flavus* [75]. In this study, the expression level of Aradu.0GH1S.1 in susceptible genotype was significantly higher than that in resistant genotype, which might be caused by the regulation of miR156. In addition, GO and KEGG results showed that the GO term and pathway related with secondary metabolism of flavonoids was enriched between two peanut varieties. These results provided valuable information for understanding the roles of miR156/SPL in peanut.

## Conclusion

**Fig. 8:**
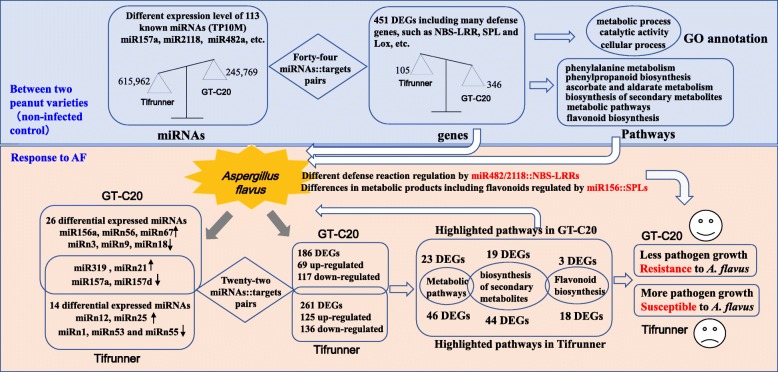
Flow chart for analysis of the transcriptome, miRNAome and degradome of R and S genotype peanut varieties in response to *A. flavus*. The gray background (top of figure 8) showed the comparison between two peanut varieties. The faint yellow background (bottom of figure 8) showed the comparison between control and treatment by *A. flavus*

Our study reported the integration analysis of the transcriptome, miRNAome and degradome of resistant and susceptible peanut varieties in response to *A. flavus*. A total of 30 differentially expressed miRNAs, 447 differentially expressed genes and 21 miRNA/targets pairs were identified in response to *A. flavus*. In addition, a total 62 differentially expressed miRNAs, 451 differentially expressed genes and 44 miRNA/targets pairs were identified between resistant and susceptible peanut varieties. Furthermore, the function of two miRNA/targets regulation pairs were discussed including miR482/2118/NBS-LRR and miR156/157/SPL (Fig. 8). Our study generated a comprehensive dataset for further understand the roles of miRNA in response to *A. flavus* in peanut.

## Methods

### Plant material and treatment condition

Two cultivated peanuts, including GT-C20 and Tifrunner, were obtained from Shandong Center of Crop Germplasm Resources, Jinan, China, and the voucher specimens were deposited in the Shandong Center of Crop Germplasm Resources. The resistance of GT-C20 and Tifrunner to *A. flavus* has ever been reported in previous studies [76]. The *A. flavus* strain (AF-Wh2014) isolated from peanut was kindly provided by lab of Prof. Boshou Liao, Oil Crops Research Institute of Chinese Academy of Agricultural Sciences. The method for evaluating the resistance of the two peanut varieties in response to *A. flavus* referred to the previous studies and was improved in this study [9, 10, 37]. Briefly, *A. flavus* was cultured in fresh potato dextrose agar (PDA) medium at 30 °C for 7 d. The conidia were then collected and suspended into spores suspension (10^6^ CFU/ml) with sterile distilled water. For inoculation with *A. flavus*, peanut seeds were surface sterilized with 70% alcohol for 1 min, and 3% sodium hypochlorite (NaClO) for 10 min. Then, the seed coat was wounded with an area of 6 mm × 5 mm. The wounded area was covered with the same size of a piece of cotton, and 20 μl of spores suspension was added on the cotton. For the control sample, 20 μl sterile water was added. To keep the humidity, the infected seeds were placed in the petri dish with two layers of filter paper wet with 5 ml sterilized water. Finally, seeds were placed into incubator at 30 °C in darkness. Evaluation of peanut resistance to *A. flavus* was performed as described in a previous study [37]. Infection index was calculated based coverage of *A. flavus* mycelium in the testa of peanut seed. Peanut interaction with *A. flavus* was evaluated using the following criteria: high resistant (infection index < 5.0), moderate resistance (infection index ≥5.0 and < 10.0), moderate susceptible (infection index ≥10.0 and < 30.0), susceptible (infection index ≥30.0 and < 50.0).

### Libraries construction and deep sequencing

Seeds of GT-C20 and Tifrunner were inoculated with *A. flavus* for 2 d for small RNA, transcriptome and degradome libraries construction. Un-inoculated seeds were used as control. For each sample, three individually biological replicates were prepared. Total RNA was isolated using Trizol Reagent (Invitrogen, USA) according to the manufacturer’s protocol. RNA quality and integrity was evaluated using electrophoresis on a 1% agarose gel and BioSpectrometer fluorescence (Eppendorf, GER). Then, the total RNAs were used for constructing small RNA, transcriptome and degradome libraries using the methods as described in previous studies, respectively [35, 36, 40, 77]. In brief, for constructing the transcriptome library, the total RNA was purified to obtain the mRNA using oligo (dT) magnetic beads. Then, the mRNA was fragmented into short fragments (about 200 bp) and further used for synthesizing the first strand of cDNA using random hexamer-primer. Next, the second-strand cDNA was synthesized using the buffer, dNTPs, RNase H and DNA polymerase I and then ligated to sequencing adapters. Following the cDNA fragments are enriched by PCR amplification. Finally, the library products are ready for sequencing. For constructing small RNA library, the total RNA was first used for purifying of small RNA molecules (18–30 nt) and then ligated of a pair of adaptors to 5′ and 3′ ends. Following, the sequencing library was prepared through reverse transcription and PCR amplification. For constructing degradome library, following steps were performed. First, mRNA was used as input RNA and annealing with Biotinylated Random Primers. Second, Strapavidin capture of RNA fragments through Biotinylated Random Primers. Third, 5′ adaptor ligation to only those RNAs containing 5-monophosphates. Finally, Reverse transcription and PCR amplification were performed. Before sequencing, the QC step was performed to qualify and quantify of the sample library using Agilent 2100 Bioanaylzer and ABI StepOnePlus Real-Time PCR System. High-throughput sequencing of small RNA, transcriptome and degradome was performed in BGI (Shenzen, China) using Illumina HiSeq2000 platform.

### MiRNA identification and bioinformatics analysis

Firstly, raw reads were cleaned up by removing low quality reads, 5′ primer contaminants, reads without 3′ primer, reads with poly A, and reads shorter than 18 nt. The clean reads were aligned by BLAST against Rfam database (version 11.0, http://rfam.janelia.org/) [78, 79] and GenBank noncoding RNA database to remove rRNA, scRNA, snoRNA, snRNA, and tRNA. Then, the rest of reads were aligned with mature miRNAs in miRbase (Version 21, http://www.mirbase.org/) [80, 81] to identify the known miRNAs. Novel miRNAs were identified according to the reported method [39]. The clean reads were first mapped to whole genome sequences of *A. duranensis* and *A. ipaensis* (https://peanutbase.org/). Then, the hairpin structures of miRNA precursors were predicted using software Minreap (http://sourceforge.net/projects/mireap) according to the following parameters. The candidate sequences could form a proper secondary hairpin, where mature miRNAs mapped region was with a size of 18–25 nt, and the distance between miRNA and miRNA* is 16–300 nt. The maximal free energy allowed to form a hairpin structure is − 18 kcal/mol [40]. The gene expression level of miRNAs from different sRNA libraries was normalized using TP10M (tags per ten million reads). Differentially expressed miRNAs between different samples were identified using R package DESeq (http://bioconductor.org/packages/DEGseq/) under the criteria of adjusted *p* value (padj) < 0.05 and fold change ≥1.5 [82].

### Degradome sequencing and data analysis

To identify the potential targets regulated by miRNAs, equal amounts of RNAs from control (TC1, TC2, GC1 and GC2) and treatment samples (TT1, TT2, GT1 and GT2) of resistant and susceptible genotypes were mixed together for degradome library construction and deep sequencing. Through preprocessing, clean tags are generated. Then, clean tags were classified by alignment with Genbank, Rfam database, and miRNA database. Next, the reads were mapped to the transcriptome data of *A. duranensis* and *A. ipaensis* using SOAP2 program with allowing only two mismatches [83]. The transcriptome data of *A. duranensis* and *A. ipaensis* were downloaded from peanutbase (https://peanutbase.org). The sense strand of peanut cDNA was used to predict miRNA cleavage sites using CLeaveLand pipeline [84]. Based on the signature number and abundance of cleaved position at each occupied transcript, the cleaved transcripts could be categorized into five categories (0, 1, 2, 3 and 4) [43].

### Transcriptome sequencing and data analysis

To analyze the expression profiles of the target genes, eight independent transcriptome libraries were sequenced. After sequencing, the adaptor sequences, low-quality reads, and empty reads were first removed. Then, the clean reads were aligned with the genome sequences of *A. duranensis* and *A. ipaensis* (https://peanutbase.org/) using SOAP2 program [83]. The gene expression level was calculated using FPKM (expected number of fragments per kilobase of transcript sequence per millions base pairs sequenced) method. The relative gene expression level between different samples was calculated using log_2_ ratio method. Differential expression genes (DEGs) between two samples were identified with the criteria of probability ≥0.8 and |log_2_(fold change)| ≥ 1 using NOIseq method [85]. To identify the putative biological functions and pathways of the target genes and DEGs, Gene Ontology (GO) annotation and KEGG (Kyoto Encyclopedia of Genes and Genomes) pathway analysis were conducted as described previously [77, 86, 87].

### Expression validation of miRNA and mRNA using qRT-PCR

To validate the high-throughput sequencing results of miRNAs and target genes, quantitative RT-PCR (qRT-PCR) was performed on ABI 7500 Real-Time PCR System (Applied Biosystems). Three biological replicates were prepared for each sample. For miRNA expression analysis, stem-loop RT-PCR method was used according to the method as described previously [88]. For target genes, primers were designed using primer premier 5.0 software (www.premierbiosoft.com). All primers including the stem-loop miRNA primers, reference genes and targets genes were listed in Additional file: Table S14. The relative expression level between different samples was calculated using the 2^-△△Ct^ method. Student’s T-test was performed to analyze the changes in gene expression after treated with *A. flavus* (*P < 0.05, **P < 0.01).

## Supplementary information

**Additional file 1: Figure S1.** Distinct RNA fragment categories in each library.

**Additional file 2: Figure S2.** Comparison of sequencing and qRT-PCR results for the miRNAs and genes using heatmap. Heatmap was generated by online software Morpheus (https://software.broadinstitute.org/morpheus) according to the relative expression in comparison with the control sample TC.

**Additional file 3: Figure S3.** Volcano plots showing the gene expression differences among different samples. The RPKM-normalized transcript count data sets were analysed by NOIseq: the x-axis shows the log-ratio (gene expression fold change after challenge) the y-axis the probability for each gene of being differentially expressed. The up-regulated and down-regulated genes were identified under the criterion of probability ≥0.8 and |log2(fold change)| ≥ 1.

**Additional file 4: Table S1.** Summary of small RNA reads from the individual libraries.

**Additional file 5: Table S2.** Statistical analysis of total sRNA mapped in peanut genome.

**Additional file 6: Table S3.** Known miRNA information in two peanut varieties.

**Additional file 7: Table S4.** Information of novel miRNAs identified from peanut miRNA libraries.

**Additional file 8: Table S5.** Classification analysis of reads from degradome library.

**Additional file 9: Table S6.** Categories of candidate cleaved sites.

**Additional file 10: Table S7.** Target genes of known miRNAs identification by degradome sequencing.

**Additional file 11: Table S8.** Target genes of novel miRNAs identification by degradome sequencing.

**Additional file 12: Table S9.** Summary of RNA-seq reads from the individual libraries.

**Additional file 13: Table S10.** Top sixteen KEGG pathways in Tifrunner and GT-C20 in response to AF infection.

**Additional file 14: Table S11.** Top sixteen enriched KEGG pathways between Tifrunner and GT-C20.

**Additional file 15: Table S12.** Correlation analysis of miRNAs expression profiles and their target genes in response to *A. flavus*.

**Additional file 16: Table S13.** Correlation analysis of miRNAs expression profiles and their target genes between peanut varieties.

**Additional file 17: Table S14.** Primers used in this study.

## Data Availability

The RNA-seq data in this study were available at NCBI BioProject: PRJNA438019 (https://www.ncbi.nlm.nih.gov/bioproject/PRJNA438019).

## Abbreviations

GC: GT-C20 control
GT: GT-C20 treatment
TC: Tifrunner control
TT: Tifrunner treatment
GO: Gene Ontology
KEGG: Kyoto Encyclopedia of Genes and Genomes
NBS-LRR: Nucleotide-binding site leucine-rich repeat
SPL: SQUAMOSA promoter binding protein-like
DEG: Differentially expressed genes
