# Supplementary material for: Integrated small RNA and mRNA expression profiles reveal miRNAs and their target genes in response to Aspergillus flavus growth in peanut seeds
Source: BMC Plant Biol. 2020 May 13;20:215. doi: 10.1186/s12870-020-02426-z (PMC7222326; doi:10.1186/s12870-020-02426-z)
Supplement: Supplementary file 1 — Additional file 1: Figure S1. Distinct RNA fragment categories in each library. [file 12870_2020_2426_MOESM1_ESM.pptx]

## Slide 1
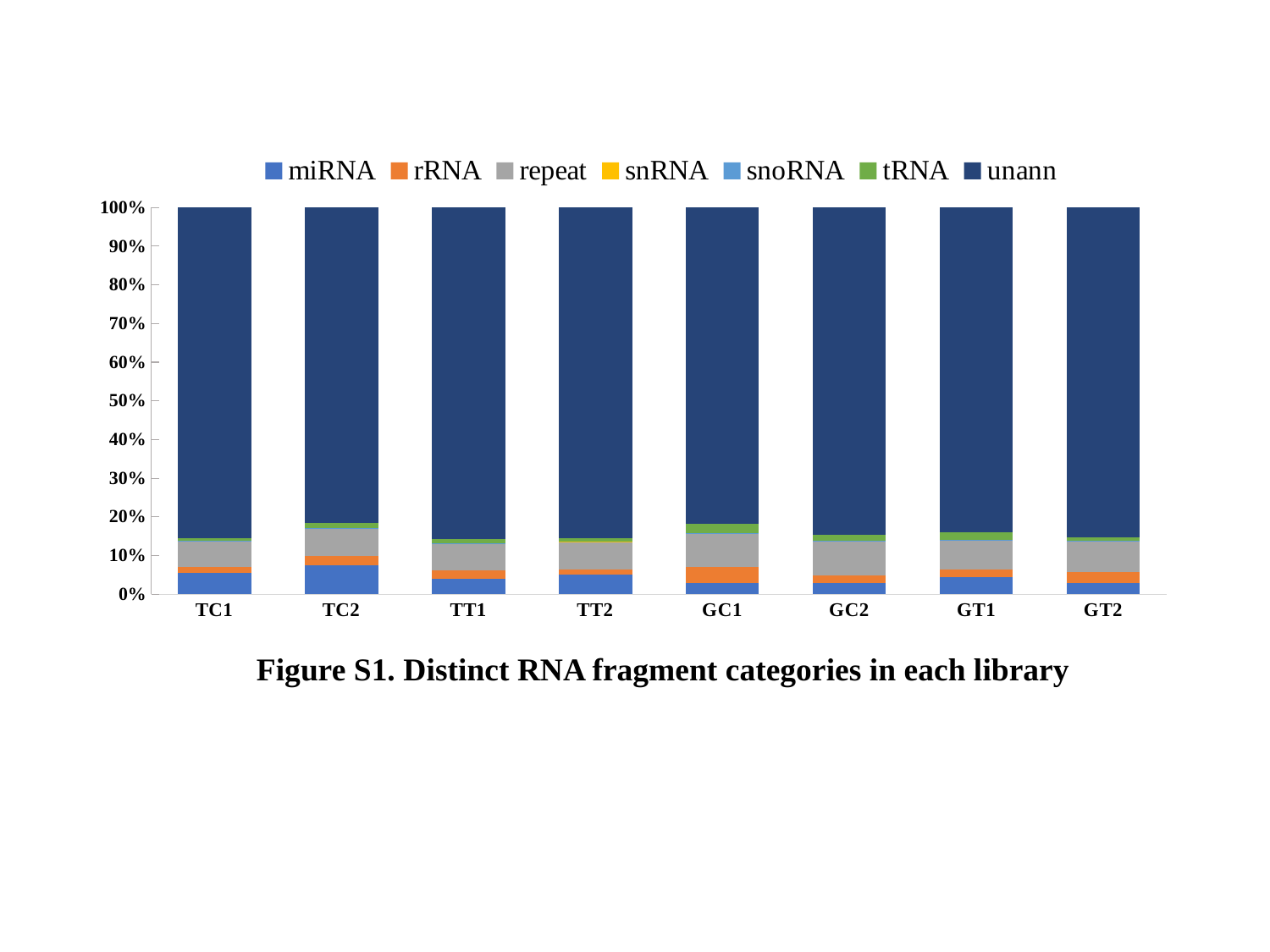

### Chart
| Category | miRNA | rRNA | repeat | snRNA | snoRNA | tRNA | unann |
|---|---|---|---|---|---|---|---|
| TC1 | 760457.0 | 204397.0 | 938094.0 | 2166.0 | 1183.0 | 98050.0 | 11818275.0 |
| TC2 | 1033628.0 | 331156.0 | 974989.0 | 2571.0 | 1306.0 | 199169.0 | 11195957.0 |
| TT1 | 534192.0 | 297730.0 | 945833.0 | 2269.0 | 1124.0 | 143966.0 | 11653011.0 |
| TT2 | 692898.0 | 164325.0 | 952523.0 | 2303.0 | 1109.0 | 114501.0 | 11438232.0 |
| GC1 | 402603.0 | 579648.0 | 1197593.0 | 3267.0 | 1647.0 | 338515.0 | 11261807.0 |
| GC2 | 406932.0 | 305019.0 | 1299182.0 | 3104.0 | 1709.0 | 223482.0 | 12313424.0 |
| GT1 | 607661.0 | 293199.0 | 1056171.0 | 3110.0 | 1696.0 | 267826.0 | 11653595.0 |
| GT2 | 416147.0 | 406705.0 | 1185732.0 | 2932.0 | 1311.0 | 146252.0 | 12438527.0 |Figure S1. Distinct RNA fragment categories in each library
