# Supplementary material for: Integrated small RNA and mRNA expression profiles reveal miRNAs and their target genes in response to Aspergillus flavus growth in peanut seeds
Source: BMC Plant Biol. 2020 May 13;20:215. doi: 10.1186/s12870-020-02426-z (PMC7222326; doi:10.1186/s12870-020-02426-z)
Supplement: Supplementary file 2 — Additional file 2: Figure S2. Comparison of sequencing and qRT-PCR results for the miRNAs and genes using heatmap. Heatmap was generated by online software Morpheus (https://software.broadinstitute.org/morpheus) according to the relative expression in comparison with the control sample TC. [file 12870_2020_2426_MOESM2_ESM.pptx]

## Slide 1
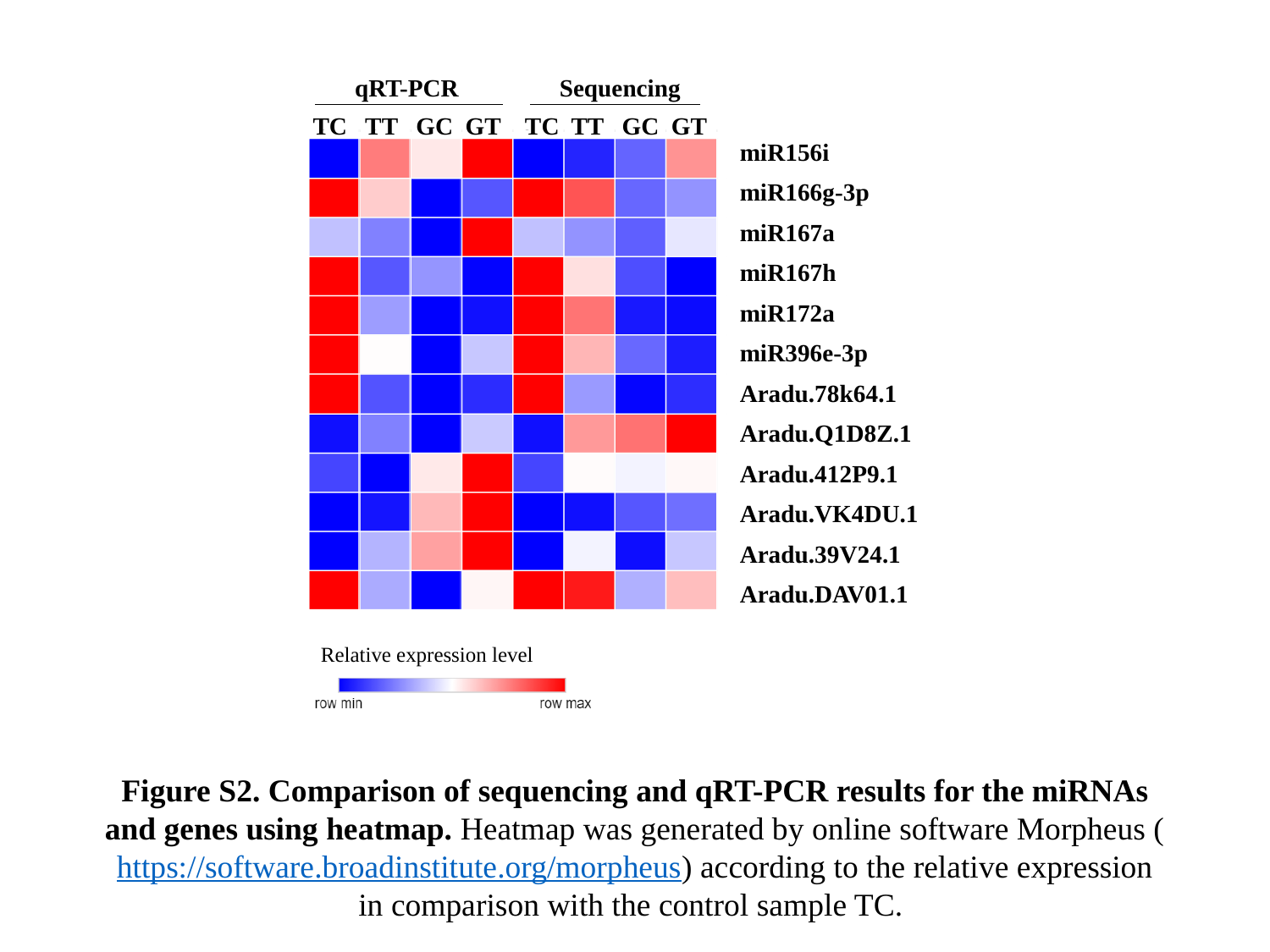

qRT-PCR
Sequencing
TC TT GC GT TC TT GC GT
Relative expression level
miR156i
miR166g-3p
miR167a
miR167h
miR172a
miR396e-3p
Aradu.78k64.1
Aradu.Q1D8Z.1
Aradu.412P9.1
Aradu.VK4DU.1
Aradu.39V24.1
Aradu.DAV01.1
Figure S2. Comparison of sequencing and qRT-PCR results for the miRNAs and genes using heatmap. Heatmap was generated by online software Morpheus (https://software.broadinstitute.org/morpheus) according to the relative expression in comparison with the control sample TC.
