# Supplementary material for: Integrated small RNA and mRNA expression profiles reveal miRNAs and their target genes in response to Aspergillus flavus growth in peanut seeds
Source: BMC Plant Biol. 2020 May 13;20:215. doi: 10.1186/s12870-020-02426-z (PMC7222326; doi:10.1186/s12870-020-02426-z)
Supplement: Supplementary file 3 — Additional file 3: Figure S3. Volcano plots showing the gene expression differences among different samples. The RPKM-normalized transcript count data sets were analysed by NOIseq: the x-axis shows the log-ratio (gene expression fold change after challenge) the y-axis the probability for each gene of being differentially expressed. The up-regulated and down-regulated genes were identified under the criterion of probability ≥0.8 and |log2(fold change)| ≥ 1. [file 12870_2020_2426_MOESM3_ESM.pdf]

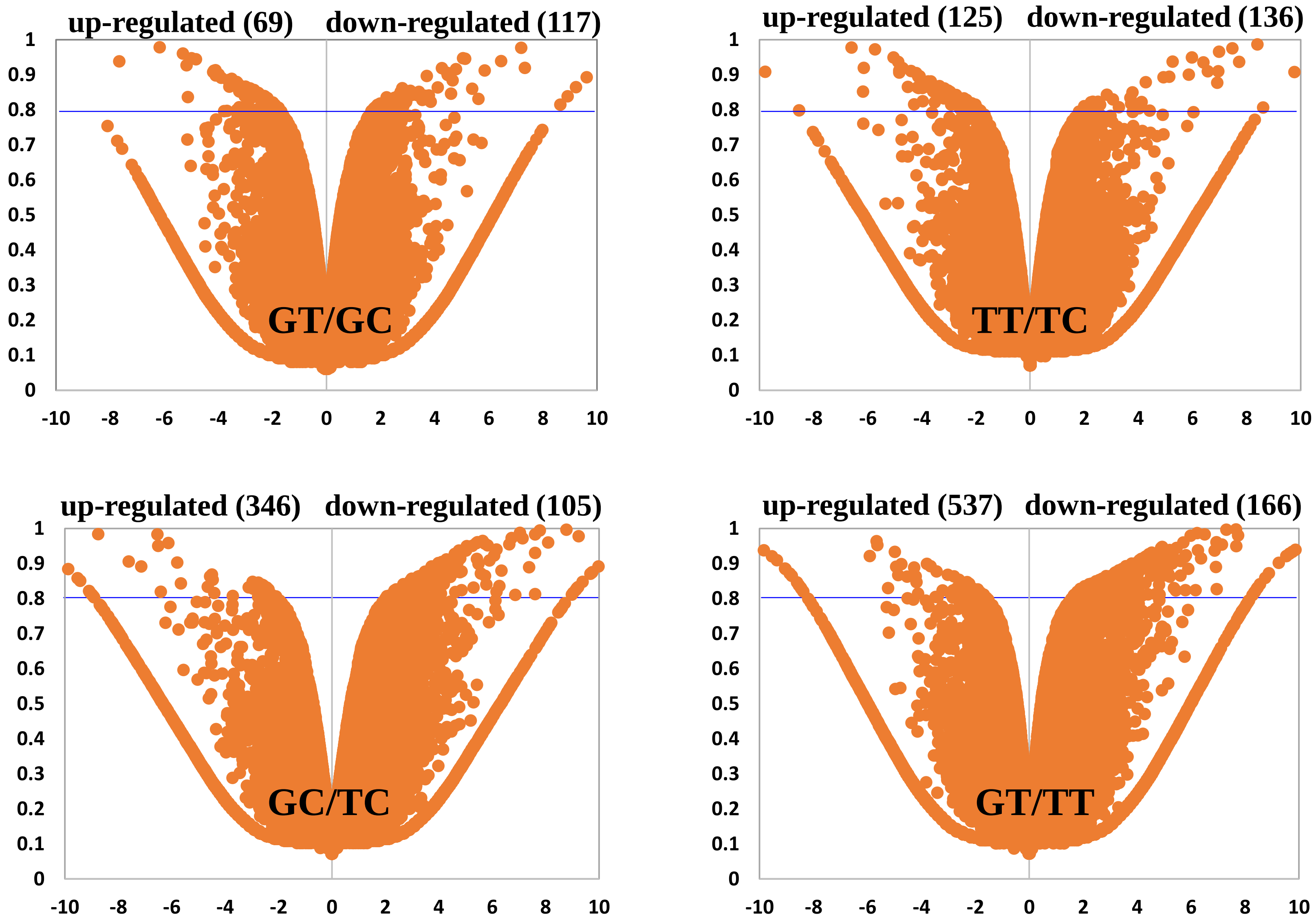

Figure S3. Volcano plots showing the gene expression differences among different samples. The RPKM-normalized transcript count data sets were analysed by using the NOIseq: the x-axis shows the log-ratio (gene expression fold change after challenge) the y-axis the probability for each gene of being differentially expressed. The up-regulated and down-regulated genes were identified under the criterion of probability ≥ 0.8 and |log2(fold change)| ≥ 1
