# Supplementary material for: Integrated small RNA and mRNA expression profiles reveal miRNAs and their target genes in response to Aspergillus flavus growth in peanut seeds
Source: BMC Plant Biol. 2020 May 13;20:215. doi: 10.1186/s12870-020-02426-z (PMC7222326; doi:10.1186/s12870-020-02426-z)
Supplement: Supplementary file 4 — Additional file 4: Table S1. Summary of small RNA reads from the individual libraries. [file 12870_2020_2426_MOESM4_ESM.docx]

**Table S1** **Summary of small RNA reads from the individual libraries**

| **Annotation** | **Reads in susceptible varieties (Tifrunner)** | | | | **Reads in resistant varieties GT-C20** | | | | | |
| --- | --- | --- | --- | --- | --- | --- | --- | --- | --- | --- |
|  | **Control 1 (TC1)** | **Control 2 (TC2)** | **Treatment 1 (TT1)** | **Treatment 2 (TT2)** | **Control 1 (GC1)** | **Control 2 (GC2)** | **Treatment 1 (GT1)** | **Treatment 2 (GT2)** | **Total** |  |
| **Total reads** | 13889088 | 13803203 | 13622803 | 13406010 | 13869544 | 14656458 | 13950577 | 14642303 | 111839986 |  |
| **3'adapter null reads** | 13552 | 13641 | 13000 | 13023 | 12096 | 13869 | 12108 | 14431 | 105720 |  |
| **Insert null reads** | 858 | 683 | 836 | 917 | 1004 | 908 | 1159 | 671 | 7036 |  |
| **5'adapter null reads** | 19002 | 22457 | 17018 | 14659 | 19007 | 25036 | 21857 | 15927 | 154963 |  |
| **Smaller than 18nt** | 30195 | 24321 | 11407 | 9096 | 50295 | 60972 | 30871 | 11241 | 228398 |  |
| **Poly A** | 2859 | 3325 | 2417 | 2424 | 2062 | 2821 | 1324 | 2427 | 19659 |  |
| **Clean reads** | 13822622 | 13738776 | 13578125 | 13365891 | 13785080 | 14552852 | 13883258 | 14597606 | 111324210 |  |
