# Supplementary material for: Integrated small RNA and mRNA expression profiles reveal miRNAs and their target genes in response to Aspergillus flavus growth in peanut seeds
Source: BMC Plant Biol. 2020 May 13;20:215. doi: 10.1186/s12870-020-02426-z (PMC7222326; doi:10.1186/s12870-020-02426-z)
Supplement: Supplementary file 5 — Additional file 5: Table S2. Statistical analysis of total sRNA mapped in peanut genome. [file 12870_2020_2426_MOESM5_ESM.docx]

**Table S2 Statistical analysis of total sRNA mapped in peanut genome**

| **Samples** | **Unique sRNAs** | **Mapped in genome** | **Percentage (%)** | **Total sRNAs** | **Mapped in genome** | **Percentage (%)** |
| --- | --- | --- | --- | --- | --- | --- |
| TC1 | 6561411 | 5379554 | 81.99% | 13822622 | 12058026 | 87.23% |
| TC2 | 6092789 | 4986291 | 81.84% | 13738776 | 12077749 | 87.91% |
| TT1 | 6438447 | 5258489 | 81.67% | 13578125 | 11677660 | 86% |
| TT2 | 6556127 | 5368433 | 81.88% | 13365891 | 11642508 | 87.11% |
| GC1 | 6714870 | 5508903 | 82.04% | 13785080 | 12066097 | 87.53% |
| GC2 | 7446546 | 6130523 | 82.33% | 14552852 | 12727475 | 87.46% |
| GT1 | 6253124 | 5098763 | 81.54% | 13883258 | 12184474 | 87.76% |
| GT2 | 7127701 | 5808762 | 81.50% | 14597606 | 12712877 | 87.09% |
