# Supplementary material for: Integrated small RNA and mRNA expression profiles reveal miRNAs and their target genes in response to Aspergillus flavus growth in peanut seeds
Source: BMC Plant Biol. 2020 May 13;20:215. doi: 10.1186/s12870-020-02426-z (PMC7222326; doi:10.1186/s12870-020-02426-z)
Supplement: Supplementary file 8 — Additional file 8: Table S5. Classification analysis of reads from degradome library. [file 12870_2020_2426_MOESM8_ESM.docx]

**Table S5 Classification analysis of reads from degradome library**

| **Classification** | **Total reads** | **Percentage** | **Unique reads** | **Percentage** |
| --- | --- | --- | --- | --- |
| Raw reads | 14338349 | 100% | 5477891 | 100% |
| Mapped in genome | 9580557 | 66.82% | 3101434 | 56.62% |
| Mapped in cDNA_sense | 5996673 | 41.82% | 1732893 | 31.63% |
| Mapped in cDNA_antisense | 274392 | 1.91% | 112985 | 2.06% |
| rRNA | 1318092 | 9.19% | 29656 | 0.54% |
| tRNA | 355 | 0.00% | 159 | 0.00% |
| snRNA | 338 | 0.00% | 214 | 0.00% |
| snoRNA | 1864 | 0.01% | 161 | 0.00% |
| PolyN | 100171 | 0.70% | 69809 | 1.27% |
| Un-annotation | 6646464 | 46.35% | 3532014 | 64.48% |
