# Supplementary material for: Integrated small RNA and mRNA expression profiles reveal miRNAs and their target genes in response to Aspergillus flavus growth in peanut seeds
Source: BMC Plant Biol. 2020 May 13;20:215. doi: 10.1186/s12870-020-02426-z (PMC7222326; doi:10.1186/s12870-020-02426-z)
Supplement: Supplementary file 9 — Additional file 9: Table S6. Categories of candidate cleaved sites. [file 12870_2020_2426_MOESM9_ESM.docx]

**Table S6** **Categories of candidate cleaved sites**

| **Categories** | **Candidate cleaved sites in all transcripts** | | **Candidate sites in matched potential**  **targets of miRNAs** | | | | |
| --- | --- | --- | --- | --- | --- | --- | --- |
|  | **Number** | **(%)** | | **Cleaved sites** | **Genes** | **miRNAs** | **Cleaved events** |
| Category 0 | 22067 | 0.79% | | 249 | 249 | 424 | 1020 |
| Category 1 | 38566 | 1.37% | | 165 | 162 | 211 | 253 |
| Category 2 | 792722 | 28.25% | | 554 | 484 | 261 | 935 |
| Category 3 | 88213 | 3.14% | | 175 | 138 | 116 | 194 |
| Category 4 | 1864429 | 66.44% | | 402 | 390 | 135 | 492 |
