# Supplementary material for: Integrated small RNA and mRNA expression profiles reveal miRNAs and their target genes in response to Aspergillus flavus growth in peanut seeds
Source: BMC Plant Biol. 2020 May 13;20:215. doi: 10.1186/s12870-020-02426-z (PMC7222326; doi:10.1186/s12870-020-02426-z)
Supplement: Supplementary file 10 — Additional file 10: Table S7. Target genes of known miRNAs identification by degradome sequencing. [file 12870_2020_2426_MOESM10_ESM.docx]

**Table [S7 Target genes of known miRNAs identification by degradome sequencing](#_Toc445656621)**

| **miRNA** | **Target gene** | **Cleavage site** | **Alignment score** | **Category** | **Function annotation** |
| --- | --- | --- | --- | --- | --- |
| ahy-miR156u | Aradu.ZPY8R.1 | 2303 | 3.5 | 0 | uncharacterized transporter |
| ahy-miR156a | Aradu.0GH1S.1 | 1139 | 2 | 2 | squamosa promoter-binding-like protein 12-like |
|  | Aradu.BF9MN.1 | 1243 | 4.5 | 2 | uncharacterized protein |
|  | Aradu.BF9MN.1 | 1243 | 4.5 | 2 | uncharacterized protein |
|  | Aradu.C99J0.1 | 123 | 4 | 2 | dnaJ homolog subfamily C member 10-like |
|  | Aradu.EH857.1 | 228 | 4.5 | 2 | unknown |
|  | Aradu.JL6EF.1 | 1151 | 4.5 | 2 | heat shock protein 83-like |
|  | Aradu.UQE92.1 | 59 | 4 | 3 | Unknown protein |
| ahy-miR156n | Aradu.0GH1S.1 | 1138 | 0 | 0 | squamosa promoter-binding-like protein 12-like |
| ahy-miR156s | Aradu.0GH1S.1 | 1138 | 1.5 | 0 | squamosa promoter-binding-like protein 12-like |
|  | Aradu.0GH1S.1 | 1139 | 1.5 | 2 | squamosa promoter-binding-like protein 12-like |
|  | Aradu.0GH1S.1 | 1139 | 1.5 | 2 | squamosa promoter-binding-like protein 12-like |
|  | Aradu.AS2MG.1 | 675 | 4.5 | 2 | splicing factor U2af large subunit B-like |
|  | Aradu.BF9MN.1 | 1243 | 4.5 | 2 | uncharacterized protein |
|  | Aradu.UQE92.1 | 58 | 4 | 2 | Unknown protein |
| ahy-miR156i | Aradu.0GH1S.1 | 1139 | 2 | 2 | squamosa promoter-binding-like protein 12-like |
| ahy-miR156f | Aradu.0GH1S.1 | 1139 | 1 | 2 | squamosa promoter-binding-like protein 12-like |
|  | Aradu.412P9.1 | 397 | 4.5 | 2 | uncharacterized protein |
|  | Aradu.6LS7N.1 | 1641 | 4 | 2 | vacuolar protein sorting-associated protein 53 homolog isoform 1 |
|  | Aradu.73JAV.1 | 1755 | 4.5 | 2 | CBL-interacting protein kinase |
|  | Aradu.NL7A8.1 | 231 | 4 | 2 | Potyvirus VPg interacting protein |
| ahy-miR156U | Aradu.0GH1S.1 | 1138 | 2 | 0 | squamosa promoter-binding-like protein 12-like |
|  |  |  |  |  |  |
| **miRNA** | **Target gene** | **Cleavage site** | **Alignment score** | **Category** | **Function annotation** |
|  | Aradu.0GH1S.1 | 1139 | 2 | 2 | squamosa promoter-binding-like protein 12-like |
|  | Aradu.0YE33.1 | 390 | 3.5 | 2 | Late embryogenesis abundant 3 (LEA3) family protein |
|  | Aradu.UQE92.1 | 58 | 4 | 2 | Unknown protein |
|  | Aradu.0GH1S.1 | 1138 | 1.5 | 0 | squamosa promoter-binding-like protein 12-like |
|  | Aradu.0GH1S.1 | 1139 | 1.5 | 2 | squamosa promoter-binding-like protein 12-like |
| ahy-miR156e | Aradu.0GH1S.1 | 1138 | 1.5 | 0 | squamosa promoter-binding-like protein 12-like |
|  | Aradu.0GH1S.1 | 1139 | 1.5 | 2 | squamosa promoter-binding-like protein 12-like |
|  | Aradu.AS2MG.1 | 675 | 4.5 | 2 | splicing factor U2af large subunit B-like |
|  | Aradu.BF9MN.1 | 1243 | 3 | 2 | profilin |
|  | Aradu.UQE92.1 | 58 | 4.5 | 2 | Unknown protein |
|  | Aradu.X9GQ3.1 | 320 | 4.5 | 2 | early nodulin-related |
| ahy-miR156r | Aradu.97TKQ.1 | 2765 | 4.5 | 2 | malate synthase, glyoxysomal |
|  | Aradu.MP8TG.1 | 763 | 4.5 | 2 | prolyl 4-hydroxylase subunit alpha-1-like |
|  | Aradu.UQE92.1 | 58 | 4 | 2 | Unknown protein |
| ahy-miR156o | Aradu.0GH1S.1 | 1139 | 1.5 | 2 | squamosa promoter-binding-like protein 12-like |
| ahy-miR156g | Aradu.0GH1S.1 | 1139 | 2 | 2 | squamosa promoter-binding-like protein 12-like |
|  | Aradu.0GH1S.1 | 1138 | 2 | 0 | squamosa promoter-binding-like protein 12-like |
| ahy-miR156h | Aradu.0GH1S.1 | 1139 | 1 | 2 | squamosa promoter-binding-like protein 12-like |
|  | Aradu.412P9.1 | 397 | 4.5 | 2 | uncharacterized protein |
|  | Aradu.6LS7N.1 | 1641 | 4 | 2 | vacuolar sorting-associated-like protein |
|  | Aradu.73JAV.1 | 1755 | 4.5 | 2 | protein kinase family protein |
|  | Aradu.NL7A8.1 | 231 | 4 | 2 | Potyvirus VPg interacting protein |
| ahy-miR156p | Aradu.0GH1S.1 | 1138 | 1.5 | 0 | squamosa promoter-binding-like protein 12-like isoform X6 |
|  |  |  |  |  |  |
| **miRNA** | **Target gene** | **Cleavage site** | **Alignment score** | **Category** | **Function annotation** |
|  | Aradu.0GH1S.1 | 1139 | 1.5 | 2 | squamosa promoter-binding-like protein 12-like isoform X6 |
| ahy-miR156l | Aradu.UQE92.1 | 59 | 4 | 3 | Unknown protein |
| ahy-miR156k | Aradu.0GH1S.1 | 1139 | 0.5 | 2 | squamosa promoter-binding-like protein 12-like isoform X6 |
| ahy-miR157k | Aradu.0GH1S.1 | 1138 | 1 | 0 | squamosa promoter-binding-like protein 12-like isoform X6 |
|  | Aradu.0GH1S.1 | 1139 | 1 | 2 | squamosa promoter-binding-like protein 12-like isoform X6 |
|  | Aradu.7N61Y.1 | 771 | 4 | 2 | plasma membrane intrinsic protein |
| ahy-miR156z | Aradu.1X7CT.1 | 45 | 4.5 | 2 | uncharacterized protein |
|  | Aradu.36E7Z.1 | 94 | 4.5 | 2 | protein DJ-1 homolog D-like isoform X2 |
|  | Aradu.B3DUX.1 | 44 | 4.5 | 2 | protein DJ-1 homolog D-like isoform X2 |
|  | Aradu.VK4DU.1 | 506 | 4.5 | 2 | peroxisomal biogenesis factor 11 family protein |
| ahy-miR160b | Aradu.39V24.1 | 1190 | 1 | 2 | auxin response factor 17 |
|  | Aradu.DXP84.1 | 1437 | 2 | 0 | auxin response factor 18-like |
|  | Aradu.M8B8F.1 | 1933 | 1 | 0 | auxin response factor 18-like |
| ahy-miR166k | Aradu.5YD67.1 | 722 | 1.5 | 2 | homeobox-leucine zipper protein REVOLUTA-like |
|  | Aradu.5YD67.1 | 722 | 1.5 | 2 | homeobox-leucine zipper protein REVOLUTA-like |
|  | Aradu.5YD67.1 | 723 | 2 | 2 | homeobox-leucine zipper protein REVOLUTA-like |
|  | Aradu.8A8RQ.1 | 1699 | 4 | 2 | Unknown protein |
|  | Aradu.BEM5C.1 | 1244 | 2 | 4 | translocase of chloroplast 90, chloroplastic-like isoform X3 |
|  | Aradu.DAV01.1 | 284 | 4.5 | 2 | peroxidase |
|  | Aradu.IH9CV.1 | 580 | 1.5 | 2 | DNA binding protein |
|  | Aradu.IH9CV.1 | 581 | 2 | 2 | DNA binding protein |
|  | Aradu.PJB9Y.1 | 69 | 1.5 | 2 | Class III homeobox-leucine zipper protein |
|  | Aradu.PJB9Y.1 | 70 | 2 | 2 | Class III homeobox-leucine zipper protein |
|  |  |  |  |  |  |
| **miRNA** | **Target gene** | **Cleavage site** | **Alignment score** | **Category** | **Function annotation** |
|  | Aradu.QWP4U.1 | 1112 | 4 | 2 | Pentatricopeptide repeat (PPR) superfamily protein |
|  | Aradu.VC8KM.1 | 254 | 1.5 | 2 | receptor-like protein kinase 4 |
|  | Aradu.VC8KM.1 | 255 | 2 | 2 | receptor-like protein kinase 4 |
| ahy-miR166e | Aradu.5YD67.1 | 721 | 3.5 | 0 | DNA binding protein |
|  | Aradu.IH9CV.1 | 579 | 3.5 | 0 | DNA binding protein |
|  | Aradu.PJB9Y.1 | 68 | 3.5 | 0 | Class III homeobox-leucine zipper protein |
|  | Aradu.VC8KM.1 | 253 | 3.5 | 0 | receptor-like protein kinase 4 |
| ahy-miR166h | Aradu.5YD67.1 | 722 | 1.5 | 2 | DNA binding protein |
|  | Aradu.5YD67.1 | 721 | 3 | 0 | DNA binding protein |
|  | Aradu.IH9CV.1 | 579 | 3 | 0 | DNA binding protein |
|  | Aradu.PJB9Y.1 | 68 | 3 | 0 | Class III homeobox-leucine zipper protein |
|  | Aradu.VC8KM.1 | 253 | 3 | 0 | receptor-like protein kinase 4 |
| ahy-miR166l | Aradu.5YD67.1 | 721 | 3 | 0 | DNA binding protein |
|  | Aradu.IH9CV.1 | 579 | 3 | 0 | DNA binding protein |
|  | Aradu.PJB9Y.1 | 68 | 3 | 0 | Class III homeobox-leucine zipper protein |
|  | Aradu.VC8KM.1 | 253 | 3 | 0 | receptor-like protein kinase 4 |
| ahy-miR169l | Aradu.JNA0U.1 | 1339 | 3.5 | 0 | nuclear transcription factor Y subunit A-9-like |
| ahy-miR169e | Aradu.0KQ9H.1 | 1191 | 2 | 4 | nuclear transcription factor Y subunit A-3-like isoform X4 |
|  | Aradu.67X2R.1 | 1051 | 2 | 4 | nuclear transcription factor Y subunit A-3-like isoform 3 |
|  | Aradu.JNA0U.1 | 1339 | 3.5 | 0 | nuclear transcription factor Y subunit A-1-like isoform X2 |
|  | Aradu.JNA0U.1 | 1339 | 2.5 | 0 | nuclear transcription factor Y subunit A-1-like isoform X2 |
|  | Aradu.X3MYC.1 | 945 | 2 | 4 | CCAAT-binding transcription factor |
| ahy-miR172a | Aradu.42K79.1 | 1431 | 2.5 | 4 | ethylene-responsive transcription factor RAP2-7-like |
|  |  |  |  |  |  |
| **miRNA** | **Target gene** | **Cleavage site** | **Alignment score** | **Category** | **Function annotation** |
| ahy-miR319e | Aradu.ML9JR.1 | 2362 | 3 | 0 | transcription factor GAMYB-like |
| ahy-miR3514-3p | Aradu.7H0DM.1 | 360 | 0.5 | 0 | pentatricopeptide repeat-containing protein |
