# Supplementary material for: Integrated small RNA and mRNA expression profiles reveal miRNAs and their target genes in response to Aspergillus flavus growth in peanut seeds
Source: BMC Plant Biol. 2020 May 13;20:215. doi: 10.1186/s12870-020-02426-z (PMC7222326; doi:10.1186/s12870-020-02426-z)
Supplement: Supplementary file 11 — Additional file 11: Table S8. Target genes of novel miRNAs identification by degradome sequencing. [file 12870_2020_2426_MOESM11_ESM.docx]

**Table [S8 Target genes of novel miRNAs identification by degradome sequencing](#_Toc445656621)**

| **miRNA** | **Target gene** | **Cleavage site** | **Alignment score** | **Category** | **Function annotation** |
| --- | --- | --- | --- | --- | --- |
| miRn1 | Aradu.32A9T.1 | 4568 | 4 | 4 | uncharacterized protein |
|  | Aradu.5S2XJ.1 | 533 | 3.5 | 2 | uncharacterized protein |
|  | Aradu.A6IZK.1 | 447 | 4.5 | 2 | uncharacterized protein |
|  | Aradu.A7MSH.1 | 4768 | 4.5 | 4 | uncharacterized protein |
|  | Aradu.DS3R5.1 | 887 | 4 | 4 | ataxin-3 homolog |
|  | Aradu.KQV51.1 | 1056 | 4.5 | 4 | ribosomal RNA processing protein 1 homolog |
| miRn4 | Aradu.BXG29.1 | 552 | 4.5 | 4 | uncharacterized protein |
|  | Aradu.NYP4Z.1 | 1759 | 4.5 | 4 | RING finger and CHY zinc finger domain-containing protein 1-like |
|  | Aradu.ZV3G2.1 | 700 | 4.5 | 4 | uncharacterized protein |
| miRn5 | Aradu.G7JI6.1 | 20 | 4.5 | 2 | hypothetical protein |
| miRn6 | Aradu.0Y40Q.1 | 1028 | 4.5 | 4 | 3-ketoacyl-CoA synthase 11-like |
|  | Aradu.3GX6J.1 | 1371 | 4, | 2 | hypothetical protein |
|  | Aradu.67AL7.1 | 305 | 4 | 4 | protein argonaute 4-like |
|  | Aradu.K63NX.1 | 190 | 2 | 4 | uncharacterized protein |
|  |  |  |  |  |  |
| **miRNA** | **Target gene** | **Cleavage site** | **Alignment score** | **Category** | **Function annotation** |
|  | Aradu.Z8HLS.1 | 2181 | 4.5 | 4 | transcriptional corepressor SEUSS-like |
| miRn7 | Aradu.8K2VK.1 | 1279 | 4.5 | 4 | RNA and export factor-binding protein 2-like |
|  | Aradu.NCP7U.1 | 1951 | 4.5 | 4 | uncharacterized protein |
| miRn8 | Aradu.12NGZ.1 | 3800 | 4.5 | 4 | protein ALWAYS EARLY 3-like |
| miRn9 | Aradu.109YL.1 | 1183 | 3.5 | 2 | unknown |
|  | Aradu.GB4V7.1 | 1159 | 4 | 4 | metal transporter Nramp3-like |
|  | Aradu.GE27A.1 | 2220 | 4.5 | 2 | uncharacterized protein |
|  | Aradu.RU1MG.1 | 1337 | 4 | 2 | ethylene receptor 2-like |
|  | Aradu.URV45.1 | 566 | 1.5 | 0 | putative pentatricopeptide repeat-containing protein |
| miRn10 | Aradu.4L72B.1 | 1452 | 4.5 | 4 | pentatricopeptide repeat-containing protein |
|  | Aradu.B3CRQ.1 | 441 | 3 | 2 | tetratricopeptide repeat protein |
|  | Aradu.H0NY1.1 | 472 | 4 | 2 | hypothetical protein |
|  | Aradu.MZL9J.1 | 1145 | 4.5 | 4 | putative G3BP-like protein-like |
|  | Aradu.S8SZ6.1 | 1604 | 4.5 | 4 | uncharacterized protein |
| miRn11 | Aradu.C5DHY.1 | 710 | 4.5 | 4 | uncharacterized protein |
|  | Aradu.FCK70.1 | 214 | 4.5 | 4 | uncharacterized protein |
|  | Aradu.FF3RE.1 | 16 | 4.5 | 4 | uncharacterized protein |
| miRn13 | Aradu.36VCP.1 | 2128 | 4.5 | 2 | Nucleoporin interacting component; Protein prenyltransferase |
|  | Aradu.BH39W.1 | 2658 | 4 | 4 | hypothetical protein |
|  | Aradu.DY6W4.1 | 2863 | 4.5 | 4 | uncharacterized protein |
|  | Aradu.GH8FF.1 | 2246 | 4 | 4 | uncharacterized protein |
| miRn14 | Aradu.28KTI.1 | 813 | 4.5 | 4 | uncharacterized protein |
|  | Aradu.3B2AS.1 | 260 | 4 | 2 | adipocyte plasma membrane-associated protein-like |
|  |  |  |  |  |  |
| **miRNA** | **Target gene** | **Cleavage site** | **Alignment score** | **Category** | **Function annotation** |
|  | Aradu.8H45I.1 | 16 | 4.5 | 4 | uncharacterized protein |
|  | Aradu.CW19Q.1 | 1335 | 4.5 | 4 | uncharacterized protein |
|  | Aradu.E26DL.1 | 1035 | 3.5 | 4 | thaumatin-like protein 1-like |
|  | Aradu.FF4D5.1 | 171 | 3.5 | 4 | Cytochrome P450 |
|  | Aradu.LBI05.1 | 721 | 4.5 | 2 | Cytochrome P450 |
|  | Aradu.M1XBW.1 | 2645 | 4 | 2 | uncharacterized protein |
|  | Aradu.NKU3K.1 | 42, | 4 | 4 | Baculoviral IAP repeat-containing protein |
|  | Aradu.Y7IVD.1 | 957 | 3.5 | 3 | seed maturation protein PM40 precurso |
| miRn15 | Aradu.0KB9D.1 | 1625 | 4.5 | 4 | AP2-like ethylene-responsive transcription factor AIL6-like |
|  | Aradu.71U9H.1 | 1603 | 4.5 | 4 | splicing factor 3B subunit 2-like |
| miRn16 | Aradu.716Q8.1 | 54 | 4.5 | 2 | JHL20J20.9 |
|  | Aradu.7NY4Q.1 | 13 | 4.5 | 4 | V-type proton ATPase subunit d2-like isoform 1 |
|  | Aradu.B90GQ.1 | 41 | 4.5 | 4 | Ethylene-responsive transcription factor |
|  | Aradu.JY9SV.1 | 497 | 4.5 | 4 | crooked neck-like protein 1-like |
| miRn17 | Aradu.9L8G9.1 | 212 | 3 | 2 | unknown |
| miRn19 | Aradu.CR2ZJ.1 | 1177 | 4 | 4 | ferrochelatase-2, chloroplastic-like |
|  | Aradu.SG50D.1 | 766 | 3.5 | 2 | hypothetical protein |
| miRn20 | Aradu.24LGE.1 | 1160 | 3.5 | 4 | SNF1-related protein kinase regulatory subunit gamma-like PV42a-like |
|  | Aradu.5H16F.1 | 218 | 3 | 3 | uncharacterized protein |
|  | Aradu.JJY9M.1 | 304 | 4.5 | 4 | probable MYST-like histone acetyltransferase 1-like |
|  | Aradu.PS85R.1 | 754 | 4 | 4 | serine/threonine-protein phosphatase PP2A catalytic subunit-like |
| miRn21 | Aradu.S2K0Q.1 | 2067 | 4.5 | 4 | dymeclin-like isoform 1 |
|  |  |  |  |  |  |
| **miRNA** | **Target gene** | **Cleavage site** | **Alignment score** | **Category** | **Function annotation** |
|  | Aradu.T4WFS.1 | 1628 | 4 | 4 | uncharacterized protein |
| miRn22 | Aradu.LK8D7.1 | 734 | 4.5 | 4 | ELMO domain-containing protein A-like |
|  | Aradu.VG5D2.1 | 298 | 4.5 | 4 | coatomer subunit delta-2-like |
| miRn24 | Aradu.7S0UL.1 | 1076 | 4.5 | 4 | serine/threonine-protein phosphatase PP1-like |
|  | Aradu.BC16K.1 | 2268 | 4 | 4 | uncharacterized protein |
| miRn25 | Aradu.6T94Y.1 | 1324 | 4.5 | 4 | ankyrin repeat-containing protein |
|  | Aradu.BP38Y.1 | 926 | 4 | 4 | Beta-glucosidase |
|  | Aradu.MV2TG.1 | 1016 | 3.5 | 4 | FH protein interacting protein FIP2-like |
|  | Aradu.SIK94.1 | 260 | 4 | 4 | uncharacterized protein |
| miRn26 | Aradu.15BWA.1 | 1844 | 4.5 | 4 | uncharacterized protein |
|  | Aradu.B0BA3.1 | 1048 | 4.5 | 4 | serine carboxypeptidase-like 12-like |
|  | Aradu.F0Y0D.1 | 217 | 4.5 | 4 | glucan endo-1,3-beta-glucosidase 1-like |
|  | Aradu.QEJ6H.1 | 1824 | 4 | 4 | uncharacterized protein |
|  | Aradu.T1Z0F.1 | 322 | 4.5 | 4 | uncharacterized protein |
|  | Aradu.U1FLK.1 | 1399 | 4.5 | 4 | hypothetical protein |
| miRn27 | Aradu.RB3TY.1 | 1210 | 3 | 4 | 2-oxoglutarate dehydrogenase |
| miRn28 | Aradu.XU3IM.1 | 443 | 4.5 | 4 | trihelix transcription factor GT-3b-like |
| miRn29 | Aradu.16W66.1 | 3501 | 4.5 | 4 | K(+) efflux antiporter 2, chloroplastic-like |
|  | Aradu.3TJ8Z.1 | 2913 | 3.5 | 4 | filament-like plant protein 6-like |
|  | Aradu.6Q428.1 | 1544 | 4.5 | 4 | sulfite reductase |
|  | Aradu.KRL7Z.1 | 365 | 4.5 | 4 | Phosphopantothenate-cysteine ligase |
|  | Aradu.P6JNB.1 | 208 | 4 | 4 | polyadenylate-binding protein 2-like |
| miRn30 | Aradu.CI289.1 | 174 | 4.5 | 0 | probable inactive receptor kinase At5g10020-like isoform 1 |
|  |  |  |  |  |  |
| **miRNA** | **Target gene** | **Cleavage site** | **Alignment score** | **Category** | **Function annotation** |
| miRn31 | Aradu.1I23V.1 | 1294 | 4.5 | 4 | sucrose synthase |
|  | Aradu.1V1I5.1 | 395 | 1.5 | 2 | abscisic acid 8&apos;-hydroxylase |
|  | Aradu.49CW6.1 | 2792 | 4.5 | 4 | anaphase-promoting complex subunit 2-like |
|  | Aradu.AIF82.1 | 813 | 4.5 | 4 | uncharacterized protein |
|  | Aradu.BD5KG.1 | 16 | 4 | 4 | peptidyl-prolyl cis-trans isomerase |
|  | Aradu.BE7PX.1 | 740 | 4.5 | 2 | kinesin-1-like |
|  | Aradu.EF37G.1 | 83 | 1 | 0 | abscisic acid 8&apos;-hydroxylase |
|  | Aradu.G7AM5.1 | 784 | 4 | 3 | 11S arachin |
|  | Aradu.HR4SC.1 | 1106 | 4.5 | 2 | uncharacterized protein |
|  | Aradu.JW82A.1 | 1188 | 4.5 | 4 | sucrose synthase-like |
|  | Aradu.VKT6C.1 | 1772 | 4.5 | 4 | cytosolic endo-beta-N-acetylglucosaminidase-like |
|  | Aradu.X4DIU.1 | 696 | 4.5 | 4 | exocyst complex component 7-like |
| miRn32 | Aradu.28KIR.1 | 1058 | 4 | 4 | bidirectional sugar transporter SWEET2-like |
| miRn34 | Aradu.6HJ87.1 | 568 | 4.5 | 2 | cytochrome P450 monooxygenase |
|  | Aradu.C3HKT.1 | 131 | 4 | 4 | uncharacterized protein |
|  | Aradu.NEB4L.1 | 730 | 3.5 | 4 | 40S ribosomal protein S3 |
| miRn36 | Aradu.623Y7.1 | 16 | 4 | 4 | pentatricopeptide repeat-containing protein |
| miRn37 | Aradu.P2S76.1 | 525 | 4.5 | 4 | Agglutinin |
| miRn38 | Aradu.5E1NU.1 | 1434 | 4.5 | 3 | lipoxygenase 1 |
|  | Aradu.CT012.1 | 6040 | 4 | 4 | uncharacterized protein |
|  | Aradu.CZ7XQ.1 | 140 | 4 | 4 | unknown |
|  | Aradu.IDD64.1 | 788 | 4.5 | 4 | CM0216.530.nc |
|  | Aradu.PC9R9.1 | 2779 | 4.5 | 4 | uncharacterized protein |
|  |  |  |  |  |  |
| **miRNA** | **Target gene** | **Cleavage site** | **Alignment score** | **Category** | **Function annotation** |
|  | Aradu.WX5KP.1 | 2680 | 4.5 | 3 | 13-lipoxygenase |
| miRn42 | Aradu.14ITD.1 | 126 | 4 | 4 | -- |
|  | Aradu.45ENN.1 | 1823 | 4.5 | 2 | CWF19-like protein 2-like |
|  | Aradu.J3TE5.1 | 1283 | 3 | 4 | uncharacterized protein |
| miRn43 | Aradu.W6H6W.1 | 777 | 4.5 | 2 | unknown |
| miRn44 | Aradu.APC82.1 | 1023 | 4.5 | 2 | F-box protein PP2-B1 |
|  | Aradu.T0IJJ.1 | 598 | 4 | 4 | uncharacterized protein |
| miRn47 | Aradu.0KI7V.1 | 1286 | 3.5 | 4 | KDEL motif-containing protein 1-like |
|  | Aradu.EBC72.1 | 1742 | 4 | 4 | hypothetical protein PRUPE_ppa010627mg |
|  | Aradu.IKV4V.1 | 308 | 4 | 4 | uncharacterized protein |
|  | Aradu.J6XYM.1 | 957 | 4 | 4 | uncharacterized protein |
| miRn48 | Aradu.M8JJ0.1 | 984 | 4.5 | 4 | uncharacterized protein |
|  | Aradu.NP275.1 | 1008 | 4.5 | 4 | uncharacterized protein |
|  | Aradu.RCS61.1 | 1258 | 4.5 | 4 | predicted protein |
|  | Aradu.Y6FFI.1 | 2919 | 4.5 | 4 | clathrin heavy chain 1-like |
|  | Aradu.ZV9U0.1 | 941 | 4 | 4 | unknown |
| miRn49 | Aradu.168L7.1 | 5318 | 4.5 | 4 | NBS-NBS-LRR type disease resistance protein |
|  | Aradu.YRC6D.1 | 783 | 4.5 | 4 | hypothetical protein |
| miRn50 | Aradu.7B7J9.1 | 1626 | 4.5 | 4 | Serine/threonine protein phosphatase |
|  | Aradu.BR5RX.1 | 2404, | 4.5 | 4 | hypothetical protein |
|  | Aradu.M7IZD.1 | 384 | 4 | 4 | uncharacterized protein |
| miRn51 | Aradu.1H4CB.1 | 3736 | 4 | 4 | inactive poly [ADP-ribose] polymerase |
|  | Aradu.512HD.1 | 269 | 4.5 | 2 | ubiquitin-like modifier-activating enzyme |
|  |  |  |  |  |  |
| **miRNA** | **Target gene** | **Cleavage site** | **Alignment score** | **Category** | **Function annotation** |
|  | Aradu.56DPU.1 | 475 | 4.5 | 4 | eukaryotic translation initiation factor |
|  | Aradu.JRF6F.1 | 332 | 4.5 | 4 | uncharacterized protein |
|  | Aradu.N1VYM.1 | 22 | 4 | 1 | unknown |
|  | Aradu.Q88CS.1 | 311 | 4.5 | 2 | F-box protein SKIP23-like |
|  | Aradu.SR1SP.1 | 522 | 4.5 | 4 | -- |
|  | Aradu.YL6AN.1 | 576 | 4 | 4 | INO80 complex subunit |
| miRn52 | Aradu.XNA23.1 | 1486 | 4.5 | 4 | nipped-B-like protein-like |
| miRn53 | Aradu.61FU3.1 | 2016 | 4 | 4 | putative uncharacterized protein |
|  | Aradu.C2I2I.1 | 2456 | 4 | 4 | uncharacterized protein |
|  | Aradu.G6U2W.1 | 466 | 4.5 | 0 | serine/threonine-protein phosphatase |
|  | Aradu.IR0WX.1 | 843 | 3.5 | 4 | tRNA-specific 2-thiouridylase mnmA-like |
|  | Aradu.SNY5Z.1 | 54 | 4.5 | 4 | -- |
|  | Aradu.T8J76.1 | 1675 | 4.5 | 4 | PREDICTED: queuine tRNA-ribosyltransferase-like |
|  | Aradu.UU57Q.1 | 980, | 4.5 | 2 | LOW QUALITY PROTEIN: cysteine proteinase 15A-like |
|  | Aradu.X5PRK.1 | 234 | 4.5 | 4 | nonclathrin coat protein zeta1-COP |
|  | Aradu.Y7C7J.1 | 84 | 4.5 | 4 | CAX-interacting protein 4-like isoform 1 |
| miRn54 | Aradu.2YF2R.1 | 629 | 3.5 | 2 | uncharacterized protein |
|  | Aradu.5113I.1 | 44 | 4.5 | 4 | probable polyol transporter 6-like |
|  | Aradu.EF42L.1 | 6251 | 4.5 | 4 | nuclear-pore anchor-like |
|  | Aradu.JT1JK.1 | 1214 | 4.5 | 2 | medium-chain-fatty-acid--CoA ligase-like |
|  | Aradu.JV10F.1 | 540 | 4.5 | 3 | elongation factor 1 beta |
|  | Aradu.K4NXS.1 | 1369 | 2 | 2 | aspartic proteinase-like |
|  | Aradu.S0XAG.1 | 1038 | 4.5 | 2 | putative oxidoreductase C1F5.03c-like |
|  |  |  |  |  |  |
| **miRNA** | **Target gene** | **Cleavage site** | **Alignment score** | **Category** | **Function annotation** |
|  | Aradu.TIE6F.1 | 262 | 4 | 2 | peroxisomal membrane protein PEX14-like |
|  | Aradu.XT8CD.1 | 801 | 4.5 | 4 | UPF0061 protein |
| miRn55 | Aradu.B9QGP.1 | 1397 | 4.5 | 4 | BZIP transcription factor bZIP39 |
| miRn58 | Aradu.217QF.1 | 336 | 4.5 | 2 | probable cyclic nucleotide-gated ion channel 20 |
|  | Aradu.3C9Y1.1 | 3914 | 4.5 | 4 | splicing factor 3B subunit 1-like |
|  | Aradu.GVC2W.1 | 1755 | 4.5 | 2 | importin subunit alpha-1-like |
|  | Aradu.M98HK.1 | 1142 | 4.5 | 4 | probable serine/threonine-protein kinase |
|  | Aradu.QZS0Y.1 | 480 | 4.5 | 4 | U-box domain-containing protein |
| miRn59 | Aradu.Y7IVD.1 | 1413 | 3.5 | 2 | seed maturation protein PM40 precursor |
| miRn61 | Aradu.08WSJ.1 | 365 | 4.5 | 4 | cysteine proteinase RD21a-like |
|  | Aradu.0K8SM.1 | 3021 | 4.5 | 4 | RNA-dependent RNA polymerase 2-like |
|  | Aradu.9RA3K.1 | 2185 | 4 | 4 | subtilisin-like protease-like |
|  | Aradu.B4FEF.1 | 1296 | 4.5 | 2 | uncharacterized protein |
|  | Aradu.BKR3K.1 | 201 | 4.5 | 4 | unnamed protein product |
|  | Aradu.FIU9K.1 | 252 | 4.5 | 4 | Homeobox-leucine zipper protein |
|  | Aradu.T9WVT.1 | 708 | 4 | 2 | uncharacterized protein |
| miRn63 | Aradu.U2CVT.1 | 2218 | 4.5 | 4 | uncharacterized protein |
|  | Aradu.UD5N8.1 | 1231 | 4 | 4 | zinc finger CCCH domain-containing protein 43-like |
|  | Aradu.V6MKI.1 | 1545 | 4.5 | 4 | pyruvate kinase, cytosolic isozyme-like |
|  | Aradu.WED0E.1 | 735 | 4.5 | 2 | pentatricopeptide repeat-containing protein |
|  | Aradu.Z6FIB.1 | 1213 | 4.5 | 2 | Pentatricopeptide repeat-containing protein |
| miRn66 | Aradu.XX5Y6.1 | 511 | 4.5 | 4 | uncharacterized protein |
| miRn67 | Aradu.33Q41.1 | 1489 | 4 | 2 | uncharacterized protein |
|  |  |  |  |  |  |
| **miRNA** | **Target gene** | **Cleavage site** | **Alignment score** | **Category** | **Function annotation** |
|  | Aradu.41QII.1 | 8843 | 4 | 2 | E3 ubiquitin-protein ligase |
|  | Aradu.Y4863.1 | 3828 | 4.5 | 4 | uncharacterized protein |
