# Supplementary material for: Integrated small RNA and mRNA expression profiles reveal miRNAs and their target genes in response to Aspergillus flavus growth in peanut seeds
Source: BMC Plant Biol. 2020 May 13;20:215. doi: 10.1186/s12870-020-02426-z (PMC7222326; doi:10.1186/s12870-020-02426-z)
Supplement: Supplementary file 13 — Additional file 13: Table S10. Top sixteen KEGG pathways in Tifrunner and GT-C20 in response to AF infection. [file 12870_2020_2426_MOESM13_ESM.docx]

**Table S10 Top sixteen KEGG pathways in Tifrunner and GT-C20 in response to AF infection**

| **Pathway** | **Gens and percentage with pathway annotation in Tifrunner** | | | | **Genes and percentage with pathway annotation in GT-C20** | | | | |
| --- | --- | --- | --- | --- | --- | --- | --- | --- | --- |
|  | **DEGs (85)** | **All genes (16649)** | **P-value** | **Q-value** | | **DEGs (51)** | **All genes (16649)** | **P-value** | **Q-value** |
| Biosynthesis of secondary metabolites | 44 (51.76%) | 2198 (13.20%) | 1.70E-17 | 9.03E-16 | | 19 (37.25%) | 2198 (13.20%) | 1.29E-05 | 0.000529867 |
| Flavonoid biosynthesis | 18 (21.18%) | 283 (1.70%) | 3.55E-15 | 9.40E-14 | | 3 (5.88%) | 283 (1.70%) | 0.05570086 | 0.216891879 |
| Stilbenoid, diarylheptanoid and gingerol biosynthesis | 13 (15.29%) | 239 (1.44%) | 2.35E-10 | 4.15E-09 | | 2 (3.92%) | 239 (1.44%) | 0.1661959 | 0.358633258 |
| Metabolic pathways | 46 (54.12%) | 4387 (26.35%) | 4.87E-08 | 6.46E-07 | | 23 (45.10%) | 4387 (26.35%) | 0.002936124 | 0.040127028 |
| Limonene and pinene degradation | 9 (10.59%) | 190 (1.14%) | 5.30E-07 | 5.62E-06 | | 2 (3.92%) | 190 (1.14%) | 0.1150129 | 0.2962619 |
| Glycolysis / Gluconeogenesis | 9 (10.59%) | 238 (1.43%) | 3.43E-06 | 3.03E-05 | | 5 (9.80%) | 238 (1.43%) | 0.000786858 | 0.016130595 |
| Isoflavonoid biosynthesis | 5 (5.88%) | 55 (0.33%) | 8.77E-06 | 6.10E-05 | | 1 (1.96%) | 55 (0.33%) | 0.1555011 | 0.358633258 |
| Flavone and flavonol biosynthesis | 7 (8.24%) | 145 (0.87%) | 9.20E-06 | 6.10E-05 | | 3 (5.88%) | 145 (0.87%) | 0.009931206 | 0.07283445 |
| Benzoxazinoid biosynthesis | 5 (5.88%) | 69 (0.41%) | 2.68E-05 | 1.58E-04 | | 4 (7.84%) | 390 (2.34%) | 0.021572381 | 0.041730021 |
| Circadian rhythm - plant | 7 (8.24%) | 195 (1.17%) | 6.20E-05 | 3.28E-04 | | 1 (1.96%) | 195 (1.17%) | 0.452158 | 0.514957722 |
| Fructose and mannose metabolism | 5 (5.88%) | 126 (0.76%) | 0.000463596 | 2.23E-03 | | 1 (1.96%) | 126 (0.76%) | 0.3216027 | 0.446304347 |
| Phenylpropanoid biosynthesis | 7 (8.24%) | 390 (2.34%) | 0.003777748 | 1.67E-02 | | 4 (7.84%) | 390 (2.34%) | 0.03123874 | 0.160098543 |
| Galactose metabolism | 4 (4.71%) | 145 (0.87%) | 0.006478984 | 2.64E-02 | | 1 (1.96%) | 145 (0.87%) | 0.3603221 | 0.447672912 |
| Pentose and glucuronate interconversions | 4 (4.71%) | 225 (1.35%) | 0.02811177 | 1.00E-01 | | 1 (1.96%) | 225 (1.35%) | 0.5009163 | 0.540462324 |
| Diterpenoid biosynthesis | 3 (3.53%) | 129 (0.77%) | 0.02833085 | 1.00E-01 | | 1 (1.96%) | 129 (0.77%) | 0.3278656 | 0.446304347 |
| Glycerolipid metabolism | 3 (3.53%) | 136 (0.82%) | 0.0324112 | 1.07E-01 | | 1 (1.96%) | 136 (0.82%) | 0.3422593 | 0.446304347 |
