# Supplementary material for: Integrated small RNA and mRNA expression profiles reveal miRNAs and their target genes in response to Aspergillus flavus growth in peanut seeds
Source: BMC Plant Biol. 2020 May 13;20:215. doi: 10.1186/s12870-020-02426-z (PMC7222326; doi:10.1186/s12870-020-02426-z)
Supplement: Supplementary file 14 — Additional file 14: Table S11. Top sixteen enriched KEGG pathways between Tifrunner and GT-C20. [file 12870_2020_2426_MOESM14_ESM.docx]

**Table S11 Top sixteen enriched KEGG pathways between Tifrunner and GT-C20**

| **Pathway** | **Gens and percentage with pathway annotation in control samples** | | | | **Genes and percentage with pathway annotation in treatment samples** | | | | |
| --- | --- | --- | --- | --- | --- | --- | --- | --- | --- |
|  | **DEGs (121)** | **All genes (16649)** | **P-value** | **Q-value** | | **DEGs (176)** | **All genes (16649)** | **P-value** | **Q-value** |
| Phenylpropanoid biosynthesis | 20 (16.53%) | 390 (2.34%) | 6.18E-12 | 3.46E-10 | | 18 (10.23%) | 390 (2.34%) | 1.73E-07 | 6.76E-06 |
| Phenylalanine metabolism | 11 (9.09%) | 169 (1.02%) | 4.14E-08 | 1.16E-06 | | 10 (5.68%) | 169 (1.02%) | 1.27E-05 | 3.29E-04 |
| Biosynthesis of secondary metabolites | 37 (30.58%) | 2198 (13.20%) | 4.70E-07 | 8.77E-06 | | 50 (28.41%) | 2198 (13.20%) | 6.94E-08 | 5.41E-06 |
| Alanine, aspartate and glutamate metabolism | 4 (3.31%) | 98 (0.59%) | 0.005671216 | 7.77E-02 | | 5 (2.84%) | 98 (0.59%) | 0.003837294 | 3.78E-02 |
| Phagosome | 5 (4.12%) | 164 (0.99%) | 0.006935562 | 7.77E-02 | | 4 (2.27%) | 164 (0.99%) | 0.0962872 | 2.78E-01 |
| Metabolic pathways | 44 (36.36%) | 4387 (26.35%) | 0.00957298 | 8.93E-02 | | 67 (38.07%) | 4387 (26.35%) | 0.000411175 | 6.41E-03 |
| Cyanoamino acid metabolism | 4 (3.31%) | 120 (0.72%) | 0.01142225 | 9.14E-02 | | 6 (3.41%) | 120 (0.72%) | 0.001732043 | 2.25E-02 |
| Flavonoid biosynthesis | 6 (4.96%) | 283 (1.70%) | 0.01731545 | 1.21E-01 | | 8 (4.55%) | 283 (1.70%) | 0.01057183 | 5.89E-02 |
| alpha-Linolenic acid metabolism | 3 (2.48%) | 91 (0.55%) | 0.02864758 | 1.78E-01 | | 4 (2.27%) | 91 (0.55%) | 0.01582535 | 8.23E-02 |
| Cysteine and methionine metabolism | 3 (2.48%) | 126 (0.76%) | 0.06411282 | 3.39E-01 | | 4 (2.27%) | 126 (0.76%) | 0.04477599 | 1.91E-01 |
| Natural killer cell mediated cytotoxicity | 2 (1.65%) | 58 (0.35%) | 0.06654603 | 3.39E-01 | | 2 (1.14%) | 58 (0.35%) | 0.1253228 | 3.37E-01 |
| Starch and sucrose metabolism | 7 (5.79%) | 513 (3.08%) | 0.08039264 | 3.56E-01 | | 9 (5.11%) | 513 (3.08%) | 0.094793 | 2.78E-01 |
| Ascorbate and aldarate metabolism | 3 (2.48%) | 141 (0.85%) | 0.0834492 | 3.56E-01 | | 6 (3.41%) | 141 (0.85%) | 0.003876586 | 3.78E-02 |
| Galactose metabolism | 3 (2.48%) | 145 (0.87%) | 0.08898387 | 3.56E-01 | | 6 (3.41%) | 145 (0.87%) | 0.004443276 | 3.85E-02 |
| Other glycan degradation | 3 (2.48%) | 153 (0.92%) | 0.1005005 | 3.75E-01 | | 6 (3.41%) | 153 (0.92%) | 0.005756936 | 4.08E-02 |
| Nitrogen metabolism | 1 (0.83%) | 89 (0.53%) | 0.478432 | 7.29E-01 | | 6 (3.41%) | 89 (0.53%) | 0.000360451 | 6.41E-03 |
