# Supplementary material for: Integrated small RNA and mRNA expression profiles reveal miRNAs and their target genes in response to Aspergillus flavus growth in peanut seeds
Source: BMC Plant Biol. 2020 May 13;20:215. doi: 10.1186/s12870-020-02426-z (PMC7222326; doi:10.1186/s12870-020-02426-z)
Supplement: Supplementary file 17 — Additional file 17: Table S14. Primers used in this study. [file 12870_2020_2426_MOESM17_ESM.doc]

**Table S14 Primers used in this study**

| **MiRNAs** | **Primer name** | **Sequence** |
| --- | --- | --- |
| ahy-miR156i | stem-loop RT primer | GTCGTATCCAGTGCAGGGTCCGAGGTATTCGCACTGGATACGACGTGCTCAC |
|  | forward primer | CGCGGTGACAGAAGAAAGTG |
| ahy-miR166g-3p | stem-loop RT primer | GTCGTATCCAGTGCAGGGTCCGAGGTATTCGCACTGGATACGACGAGGAATG |
|  | forward primer | TCGGACCAGGCTTCATTCCT |
| ahy-miR167a | stem-loop RT primer | GTCGTATCCAGTGCAGGGTCCGAGGTATTCGCACTGGATACGACTAGATCAT |
|  | forward primer | GTGAAGCTGCCAGCATGATCT |
| ahy-miR167h | stem-loop RT primer | GTCGTATCCAGTGCAGGGTCCGAGGTATTCGCACTGGATACGACTAAGATC |
|  | forward primer | CGTGAAGCTGCCAGCATGATC |
| ahy-miR172a | stem-loop RT primer | GTCGTATCCAGTGCAGGGTCCGAGGTATTCGCACTGGATACGACATGCAGC |
|  | forward primer | CGGAGAATCTTGATGATGCTG |
| ahy-miR396e-3p | stem-loop RT primer | GTCGTATCCAGTGCAGGGTCCGAGGTATTCGCACTGGATACGACTCTCCCAC |
|  | forward primer | GGCTCAAGAAAGCTGTGGG |
| Universal | reverse primer | TATCCAGTGCAGGGTCCGAGGTAT |
| U6 | forward primer | CATCCGATAA AATTGGAACGA |
|  | reverse primer | TTTGTGCGTGTCATCCTTGCG |
| Aradu.78K64.1 | forward primer | GCTATAGCATTGGTTGGTTCTTCAT |
|  | reverse primer | CACTTGGTGGGTGCTTGTGTCATAG |
| Aradu.Q1D8Z.1 | forward primer | GTTCACTTCCACACCGTTCTTTCAG |
|  | reverse primer | GAATACGTGGGCGCTTCAGAAAACA |
| Aradu.412P9.1 | forward primer | TCGTCATAGTTTTCTGTTGGGGTGT |
|  | reverse primer | GAAGATGATAACCCTTTTCCACCAA |
| Aradu.VK4DU.1 | forward primer | GTTTTTGAGTAATGGGGAACCTGGC |
|  | reverse primer | TCTTCGACTTTCCCAACAGAATTAG |
| Aradu.39V24.1 | forward primer | GGTTCTGGTTCATTTCTGCTGTTTG |
|  | reverse primer | CATCAAGCCTGAGCAATGTTGAGTA |
| Aradu.DAV01.1 | forward primer | TGGCTCCCAAAACTTGTTTCCTCTT |
|  | reverse primer | TGTCTTCAGCCTGAGGGCATGTCTC |
| Actin | forward primer | GTCATCGTCATCCTCTTCTC |
|  | reverse primer | CATTCCTGTTCCATTGTCAC |
